# DeepPlaque: a scalable multimodal platform for Aβ pathology and cell analysis in Alzheimer’s disease

**DOI:** 10.1038/s44321-026-00488-4

**Published:** 2026-07-21

**Authors:** Hiu Yi Wong, Cheng Jin, Sze Long Yuen, Xin Yang, Sunveer Singh Gill, Jiahui Xu, Kuo Pin Eric Lai, Wing-Yu Fu, Weina Gao, Xi Wang, Jiani Wang, Han Cao, Ge Lv, Kin Ying Mok, Ruijun Tian, Amy K Y Fu, Hao Chen, Nancy Y Ip

**Affiliations:** 1 Division of Life Science, State Key Laboratory of Nervous System Disorders, Daniel and Mayce Yu Molecular Neuroscience Center, The Hong Kong University of Science and Technology, Hong Kong Special Administrative Region, China; 2 InnoHK Hong Kong Center for Neurodegenerative Diseases, Hong Kong Science Park, Hong Kong Special Administrative Region, China; 3 Department of Computer Science and Engineering, The Hong Kong University of Science and Technology, Hong Kong Special Administrative Region, China; 4 Bayomics Biotechnology Hong Kong Limited, Hong Kong Special Administrative Region, China; 5 Shenzhen BayOmics Biotechnology Co., Ltd, Shenzhen, China; 6 Department of Neurodegenerative Disease, University College London Institute of Neurology, London, UK; 7 School of Medicine, The Hong Kong University of Science and Technology, Hong Kong Special Administrative Region, China; 8 State Key Laboratory of Medical Proteomics and Shenzhen Key Laboratory of Functional Proteomics, Department of Chemistry and Research Center for Chemical Biology and Omics Analysis, School of Science and Guangming Advanced Research Institute, Southern University of Science and Technology, Shenzhen, 518055 China; 9 Guangdong Provincial Key Laboratory of Brain Science, Disease and Drug Development; Shenzhen-Hong Kong Institute of Brain Science, HKUST Shenzhen Research Institute, Shenzhen, Guangdong China; 10 SIAT–HKUST Joint Laboratory for Brain Science, Shenzhen, Guangdong China; 11 Department of Chemical and Biological Engineering, The Hong Kong University of Science and Technology, Hong Kong Special Administrative Region, China; 12 HKUST Shenzhen-Hong Kong Collaborative Innovation Research Institute, Futian, Shenzhen, China; 13 Present Address: School of Medicine, The Hong Kong University of Science and Technology, Hong Kong Special Administrative Region, China

**Keywords:** Computational Biology, Neuroscience

## Abstract

Histological analysis is essential for understanding disease pathology and the microenvironment, particularly in Alzheimer’s disease (AD), characterized by beta-amyloid (Aβ) plaques that exist as diffuse, fibrillar, and core species, with distinct toxicity levels. However, accurate classification of Aβ plaque types in postmortem brain tissues and profiling of surrounding cells present significant challenges. To address these challenges, we developed “DeepPlaque”, an integrated system featuring “PlaqueNet”, a deep learning model for automated classification of Aβ plaque species from diverse imaging platforms. DeepPlaque includes automated workflows for cellular phenotyping and proteomic profiling through targeted laser microdissection. PlaqueNet achieves expert-level accuracy (AUC > 90%) in classifying the 3 major Aβ plaque species, supporting consistent and large-scale annotation. By integrating spatial cellular phenotyping with laser microdissection, DeepPlaque enables high-throughput proteomic analysis of Aβ plaque niches, revealing that microglia are more abundant around core and fibrillar Aβ plaques, with increased expression of apolipoprotein E and amyloid precursor protein in core Aβ plaques. This customizable platform enhances the molecular and cellular characterization of Aβ plaque-associated environments, providing critical insights into AD pathology.

## Synopsis

DeepPlaque integrates a deep learning model “PlaqueNet” with spatial phenotyping and laser microdissection proteomics. It automates the distinction of β-amyloid (Aβ) plaque species and characterizes their cellular and molecular microenvironments in Alzheimer’s disease (AD).

- PlaqueNet achieved expert-level performance (AUC > 90%) in classifying distinct Aβ plaque types — diffuse, fibrillar, and core plaques — and demonstrated strong cross-platform generalizability.
- DeepPlaque revealed an increased microglial abundance surrounding fibrillar and core plaques, and a decreased homeostatic microglial marker P2ry12 specifically in plaque-associated microglia.
- Targeted laser microdissection–based proteomic profiling identified distinct molecular signatures for each Aβ plaque species, with core plaques showed an enrichment of APOE and APP.

## Abstract (web summary)

DeepPlaque integrates a deep learning model “PlaqueNet” with spatial phenotyping and laser microdissection proteomics. It automates the distinction of β-amyloid (Aβ) plaque species and characterizes their cellular and molecular microenvironments in Alzheimer’s disease (AD).

## Graphical abstract

**Figure:**
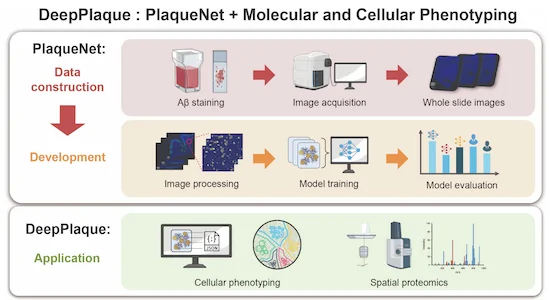

**Figure:**
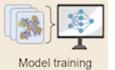

The paper explained **Problem**

Histopathological analysis of postmortem human brain tissue is crucial for understanding Alzheimer’s disease (AD) pathology and its associated microenvironment, particularly beta-amyloid (Aβ) plaque deposition. Aβ plaques exhibit diverse species, each with distinct effects on surrounding brain cells, impacting inflammation, neurodegeneration, and treatment efficacy. However, accurate identification and detailed study of these plaque types in human brain tissue are technically challenging and labor-intensive, hindering research progress.

**Results**

To address these limitations, we developed “PlaqueNet”, a deep learning model that accurately and automatically classifies diffuse, fibrillar, and core plaques from immunofluorescence-labeled whole-slide images with expert-level performance (AUC >90%). We further integrated PlaqueNet into “DeepPlaque”, a comprehensive system that combines spatial histology with laser microdissection proteomics to examine specific brain cells interacting with different Aβ plaque species. DeepPlaque enables the examination of distinct cellular interactions with Aβ plaque species and their molecular signatures. Key findings include: (1) Accurate classification for diffuse, fibrillar, and core Aβ plaques; (2) Increased microglial abundance surrounds fibrillar and core plaques, and a decrease in the homeostatic microglial marker P2ry12 specifically in plaque-associated microglia; (3) Distinct molecular signatures for each Aβ plaque species, including an enrichment of APOE and APP in core plaques.

**Impact**

This proof-of-concept study demonstrates how this AI-based tool facilitates advanced automated detection and classification of Aβ plaques, enabling robust, scalable, and accurate spatial cellular phenotyping and proteomic profiling of postmortem AD brain samples. This reduces reliance on manual interpretation, leading to a more precise characterization of disease stages. The DeepPlaque system provides a scalable framework that can be expanded to study other protein aggregates and neurodegenerative diseases, supporting more personalized and mechanism-based approaches to brain disease.

## Introduction

Histopathological analysis of postmortem human brain tissue is essential for characterizing the brain disease pathology, particularly in Alzheimer’s disease (AD). AD is a neurodegenerative condition characterized by the accumulation of extracellular beta-amyloid (Aβ) plaques and intracellular neurofibrillary tangles (Scheltens et al, 2021). Aβ deposition is a key indicator of disease progression and exists in various morphological and biochemical forms, primarily classified as diffuse, fibrillar, or dense-core (i.e., neuritic) plaques. These forms differ in structure, timing of emergence, and biological impact (Finder and Glockshuber, 2007; Dickson and Vickers, 2001; Yang et al, 2017; Sakono and Zako, 2010). The progression of Aβ plaque compaction begins with the aggregation of soluble Aβ oligomers into insoluble fibrillar Aβ plaques, ultimately forming dense-core Aβ plaques (Baik et al, 2016; Hu et al, 2009; Holtzman et al, 2000; Condello et al, 2015; Finder and Glockshuber, 2007). Diffuse Aβ plaques comprise Aβ oligomers and are toxic to synapses in the early stages of pathology, while fibrillar and dense-core Aβ plaques, which emerge later, are closely associated with synaptic dysfunction, neuroinflammation, and local neuronal injury (Gutierrez and Limon, 2022; Sakono and Zako, 2010; Dickson and Vickers, 2001; Yang et al, 2017). The burden, morphology, and spatial organization of Aβ plaques affect local cellular responses. Interactions between distinct Aβ plaque species and neighboring neurons, microglia, and astrocytes play a pivotal role in AD pathogenesis, creating unique microenvironments that affect synaptic integrity, inflammatory signaling, and disease progression (Baik et al, 2016; Holtzman et al, 2000; Hu et al, 2009; Lemke and Huang, 2022). To advance our understanding of AD, it is important to closely examine Aβ plaque microenvironments, including glia–glia interactions and neuron–microglia–astrocyte communication (Mallach et al, 2024). Understanding the spatial and functional relationships between Aβ plaques and surrounding microglia is particularly important, because microglial activation is central to amyloid sequestration and clearance (Condello et al, 2015; Yuan et al, 2016; Spangenberg et al, 2019). The efficacy of newly developed, disease-modifying, Aβ-targeting antibodies largely depends on microglial engagement and responsiveness to plaque-associated Aβ species (Swanson et al, 2021; Sims et al, 2023; van Olst et al, 2025). However, systematically assessing Aβ plaque–cell relationships across brain regions remains challenging when performed manually, limiting our ability to connect Aβ plaque niches with disease stages and cell-type-specific phenotypes.

Recent advances in digital pathology have shifted Aβ plaque analysis in histopathologically stained postmortem AD brain tissues from traditional manual assessment to automated, artificial intelligence (AI)-based approaches (Baxi et al, 2022). Deep learning models, especially convolutional neural networks, now enable large-scale detection and quantification of Aβ deposition, generally treating Aβ plaques as a single and homogeneous class. This facilitates high-throughput analysis, disease staging, and the study of Aβ plaque polymorphism and composition (Tang et al, 2019; Vizcarra et al, 2020; Wong et al, 2022; Koutarapu et al, 2025). Although these methods can effectively estimate global Aβ plaque burden, they often rely on coarse summary metrics and overlook the heterogeneity of Aβ plaque species, which exhibit diverse morphologies, variable compaction, and poorly defined boundaries. Moreover, most of these deep learning models have limited generalizability across immunostaining protocols and imaging systems. In addition, current AI workflows primarily focus on the presence or area of Aβ plaques, failing to capture their biological context. This leads to overlooked interactions with surrounding neurons, microglia, and astrocytes, as well as a lack of characterization of Aβ plaque-associated cellular microenvironments. Consequently, existing AI-based analyses have a limited ability to link the morphology and spatial organization of Aβ plaques to cellular responses, disease stages, and therapeutic mechanisms, hindering further studies of cellular mechanisms.

Accordingly, in this study, we developed a deep learning model called “PlaqueNet” for automated analysis of distinct Aβ plaques. PlaqueNet has been trained and validated on immunofluorescence-labeled Aβ plaque types, including diffuse, fibrillar, and core species, from postmortem brain sections of AD patients, achieving expert-level accuracy in classification, with an area under the curve (AUC) >90%. Furthermore, we integrated PlaqueNet into the “DeepPlaque system,” which combines image analysis and laser microdissection to identify specific brain cells that interact with different Aβ plaque species. For instance, the system enables spatial phenotyping of microglia in relation to neighboring Aβ plaques, revealing that Aβ-interacting microglia exhibit decreased levels of P2ry12, a marker of homeostatic microglia. In addition, using laser microdissection and high-throughput spatial proteomics, we performed targeted proteomic profiling of defined Aβ plaque niches, uncovering distinct molecular compositions that suggest key biological processes involved in Aβ plaque development. Thus, the DeepPlaque system serves as a scalable platform for multimodal investigation of Aβ pathology and analyses of brain cells in specific microenvironments, advancing histopathological studies of AD in postmortem brains.

## Results

### PlaqueNet accurately detects and classifies distinct species of Aβ plaques

**Figure 1:**
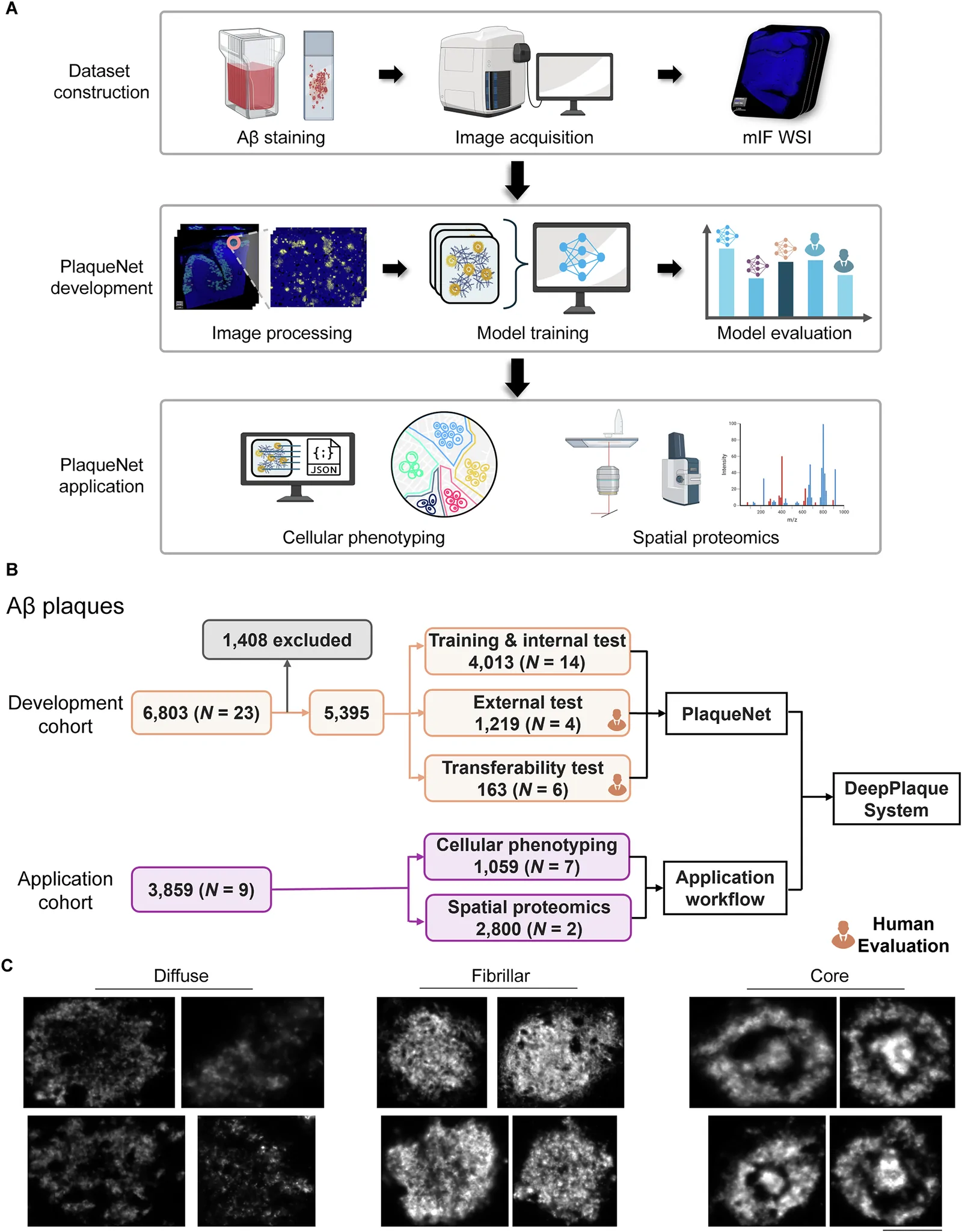
Overview of the DeepPlaque workflow. (**A**) Schematics illustrating a workflow of dataset construction using multiplex immunofluorescence (mIF) whole-slide images (WSIs) of postmortem brain sections; an outline of the development of the PlaqueNet model, including image processing, training, and model evaluation; and PlaqueNet application for cellular phenotyping and proteomics. Illustrative icons created in BioRender (Jin, C., 2025, https://BioRender.com/mz4nyrx). (**B**) Schematic diagram summarizing the development cohort for PlaqueNet (*N* = 5395 plaques from 23 brain samples) and application cohort for cellular phenotyping (*N* = 1059 plaques from seven brain samples) and spatial proteomics (*N* = 2800 plaques from two brain samples). Human evaluation was performed for external and transferability tests. (**C**) Representative images of the 3 beta-amyloid (Aβ) plaque species classified by the model (i.e., diffuse, fibrillar, and core). Scale bar = 25 µm. Source data are available online for this figure.

**Figure EV1:**
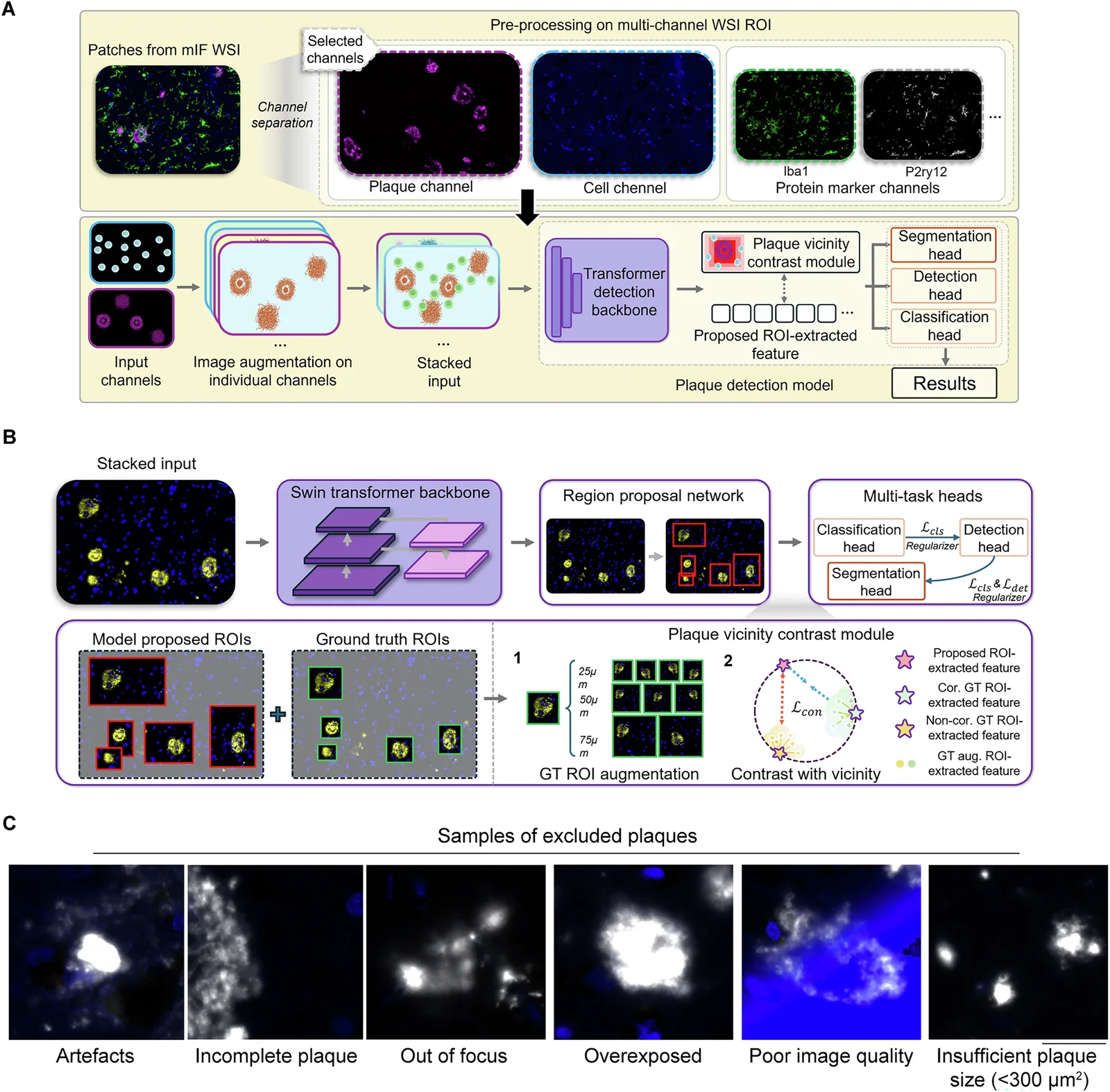
DeepPlaque preprocessing and PlaqueNet architecture. (**A**) Preprocessing of multiplex immunofluorescence (mIF) whole-slide images (WSIs), including channel separation and individual channel augmentations, leading to a stacked input into PlaqueNet. (**B**) PlaqueNet’s architecture utilizes a Swin transformer backbone and a region proposal network to feed multitask heads for classification, detection, and segmentation. (**C**) Quality control procedures and illustrations of unsatisfactory plaque samples were excluded. Aug augmented, Cor corrected, GT ground truth, ROI region of interest. Scale bar = 20 μm.

To automatically classify distinct Aβ plaque species, we developed PlaqueNet: an open-weight, deep learning model based on Swin transformer architecture (Liu et al, 2021) that is designed to segment distinct Aβ plaque species (Figs. 1A and EV1). Because of the limited amount of available training data (5395 Aβ plaques; Fig. 1B; Dataset EV1), we enhanced the model’s performance by initializing it with general image features, reducing the need for a large training set. Moreover, we employed a neighborhood‑based contrastive strategy to improve the differentiation of Aβ plaques from their surrounding microenvironment (Fig. EV1A,B). Details about sample collection, dataset construction, and model training are provided in the Methods section (Figs. 1B,C and EV1; Dataset EV1). In the PlaqueNet development cohort, we evaluated the accuracy of PlaqueNet through internal, external, and transferability tests, comparing its performance with expert evaluations (Fig. 1B). We then developed an algorithm-based workflow for studying the proteomic profiling of Aβ plaque species and the spatial cellular phenotyping of surrounding brain cells (i.e., microglia) in the application cohort using laser microdissection (Fig. 1A,B). This integration of PlaqueNet with spatial cellular phenotyping and proteomics workflow yielded the DeepPlaque system (Fig. 1).

Rigorous benchmarking against established models is essential to determine whether the performance of PlaqueNet (Fig. 1) reflects true biological signals rather than specific effects of datasets or implementation. As such, we compared PlaqueNet with several leading object detection models, including YOLOv3 (Redmon and Farhadi, 2018), RetinaNet (Lin et al, 2020), Faster R-CNN (Ren et al, 2017), and DETR (Carion et al, 2020), using data from the PhenoImager platform, which is the primary imaging system used for PlaqueNet development. Because of the limited size of this dataset, we performed threefold cross-validation for model selection and subsequently retrained PlaqueNet on the entire internal validation dataset with the optimized configuration. This strategy is commonly adopted to maximize model capacity before external evaluation (Maros et al, 2020; Tayebi Arasteh et al, 2024).

**Figure 2:**
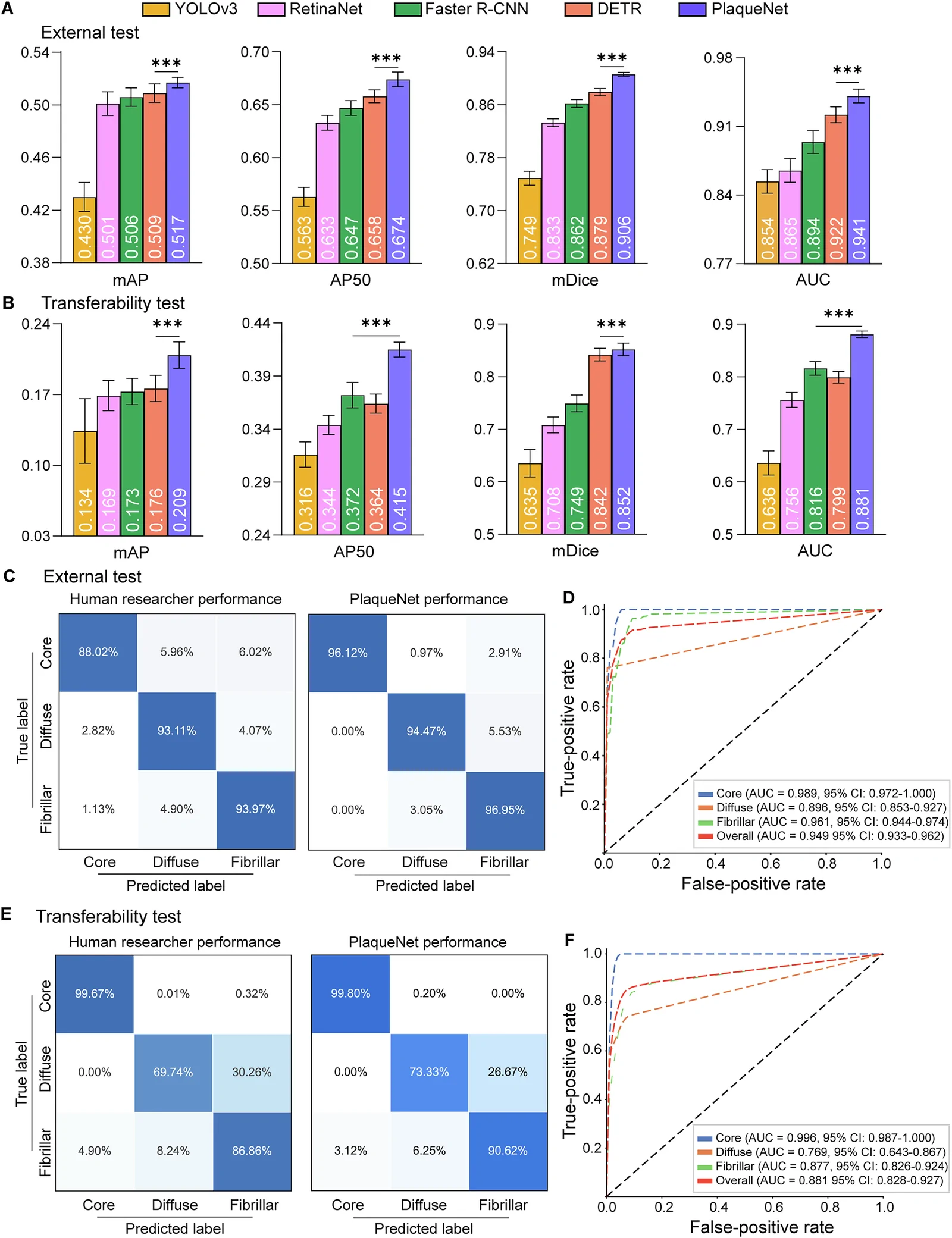
Performance evaluation of PlaqueNet. (**A**,** B**) Quantitative comparison of PlaqueNet with object detection models (i.e., YOLOv3, RetinaNet, Faster R-CNN, and DETR) across the (**A**) external validation dataset and (**B**) transferability test dataset. Performance was evaluated according to mean average precision (mAP), average precision at intersection over union (IoU) >0.50 (AP50), Dice coefficient for segmentation, and area under the curve (AUC) for classification. Numbers within histograms represent means ± SEM. Error bars are 95% confidence intervals from bootstrapping 1000 times. For (**A**), *p* = 4.19E-04 (mAP), 6.21E-06 (AP50), 5.16E-28 (mDice), and 1.41E-17 (AUC); For (**B**), *p* = 6.23E-27 (mAP), 3.36E-35 (AP50), 5.35E-04 (mDice), and 9.01E-69 (AUC); one-sided Wilcoxon signed-rank test. *p* values between all models were compared, but only *p* values comparing the best-performing method with the second-best method are shown. (**C**) Direct comparison of PlaqueNet with five expert researchers in external testing. Confusion matrices showing the classification accuracy for diffuse, fibrillar, and core Aβ plaques on the external validation dataset. (**D**) Bootstrapped receiver operating characteristic (ROC) curves and AUCs with 95% confidence intervals (CIs) for each beta-amyloid (Aβ) plaque species on the external validation dataset. (**E**) Direct comparison of PlaqueNet with five expert researchers in transferability testing. Confusion matrices showing the classification accuracy for diffuse, fibrillar, and core Aβ plaques on the transferability test dataset. (**F**) Bootstrapped receiver operating characteristic (ROC) curves and areas under the curve (AUCs) with 95% CIs for each Aβ plaque class on the transferability test dataset.

**Figure EV2:**
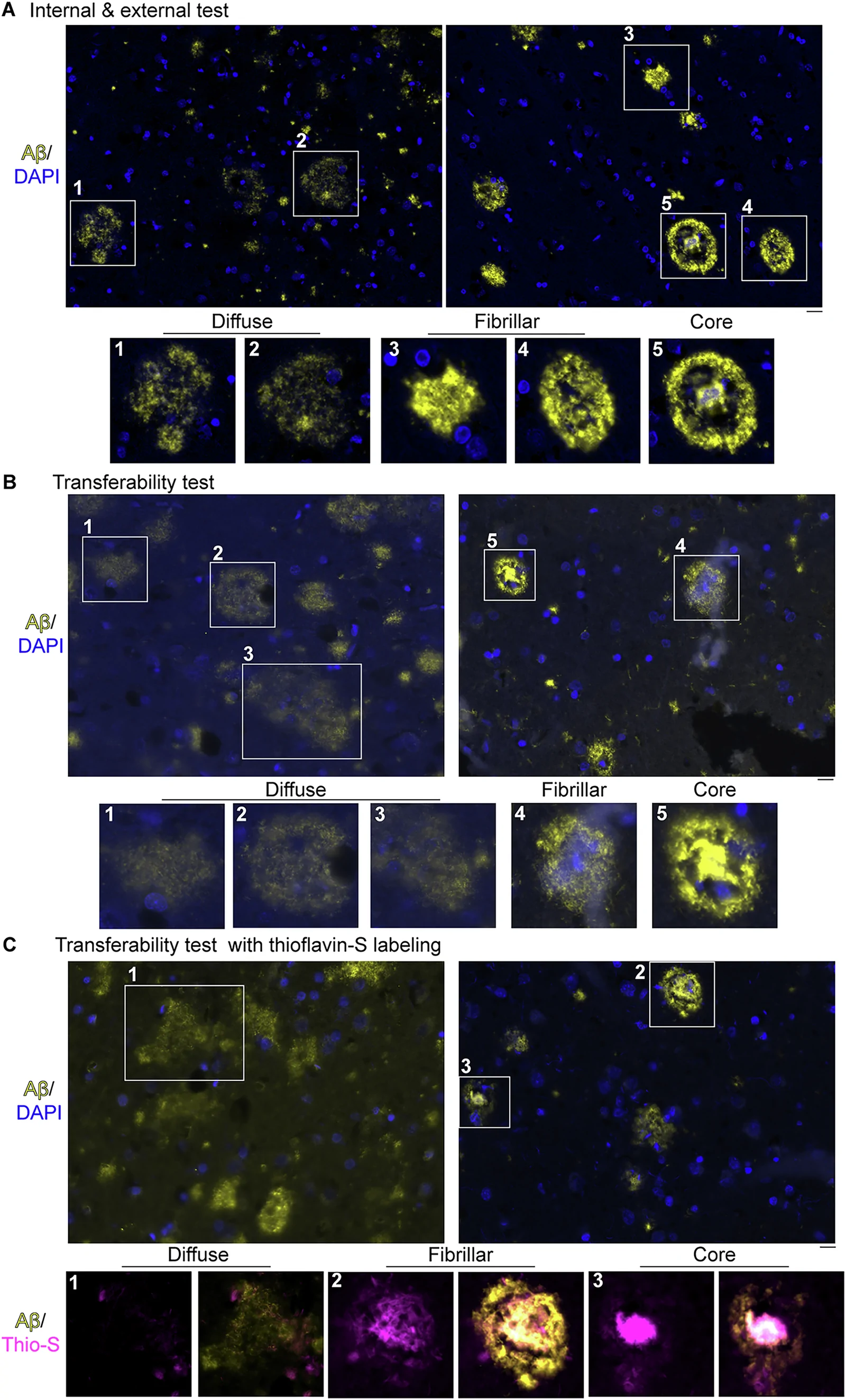
Representative examples of Aβ plaque subtypes detected by PlaqueNet. (**A**) Examples from internal and external validation tests stained for beta-amyloid (Aβ, yellow) and nuclei (DAPI, blue). (**B**) Examples of three Aβ plaque species (i.e., diffuse, fibrillar, and core) from transferability testing stained for Aβ (yellow) and nuclei (blue). (**C**) Examples of three Aβ plaque species from transferability testing stained for thioflavin-S (magenta), Aβ (yellow), and nuclei (blue). Scale bar = 20 µm.

To assess PlaqueNet, we evaluated its performance on an independent external validation dataset generated using the same imaging system for PlaqueNet development and a transferability dataset generated with a new imaging system unseen by PlaqueNet. First, to assess the generalization of PlaqueNet in newly stained AD postmortem brain samples, we evaluated its performance on an independent external validation dataset (*N* = 1219 Aβ plaques) acquired on the same imaging system (Fig. 1B). On these previously unseen biological data, PlaqueNet achieved a mean average precision (mAP) of 0.517, average precision at intersection over union (IoU) >0.50 (AP50) of 0.674, mean Dice (mDice) coefficient of 0.906, and mean classification accuracy with class-specific AUCs of 0.941 (all *p* < 0.001 comparing the best-performing model with the second-best model; Figs. 2A and EV2A). These results indicate that the model maintains a high confidence level while detecting most targets, even in the complex, low-contrast backgrounds characteristic of biological imaging. To assess the robustness of PlaqueNet on a new imaging system, we evaluated its performance on a transferability dataset (*N* = 163 Aβ plaques) acquired with the THUNDER Imager Tissue system, which was not used during PlaqueNet development (Fig. 1B). While all other models exhibited performance degradation, PlaqueNet maintained an mDice of 0.852 and an overall AUC of 0.881, significantly outperforming the next-best models (i.e., DETR and Faster R-CNN, both *p* < 0.001 comparing the best-performing model with the second-best model) (Figs. 2B and EV2B,C). This confirms PlaqueNet’s reliability for applications across different imaging systems.

Finally, we compared the performance of PlaqueNet with that of five professional researchers on both external validation and transferability datasets using ground-truth labels provided by the same expert who annotated the training images. In the external validation test, PlaqueNet achieved higher classification accuracy than the human average for diffuse Aβ plaques (94.47 vs. 93.11%), fibrillar Aβ plaques (96.95 vs. 93.97%), and core Aβ plaques (96.12 vs. 88.02%) (Fig. 2C,D). In the transferability test, PlaqueNet maintained higher classification accuracy than the human average for diffuse Aβ plaques (73.33 vs. 69.74%), fibrillar Aβ plaques (90.62 vs. 86.86%), and core Aβ plaques (99.80 vs. 99.67%) (Fig. 2E,F). Notably, the model demonstrated greater consistency and decreased confusion between ambiguous Aβ plaque species.

### DeepPlaque robustly quantifies and phenotypes the microglia that interact with distinct Aβ species

**Figure 3:**
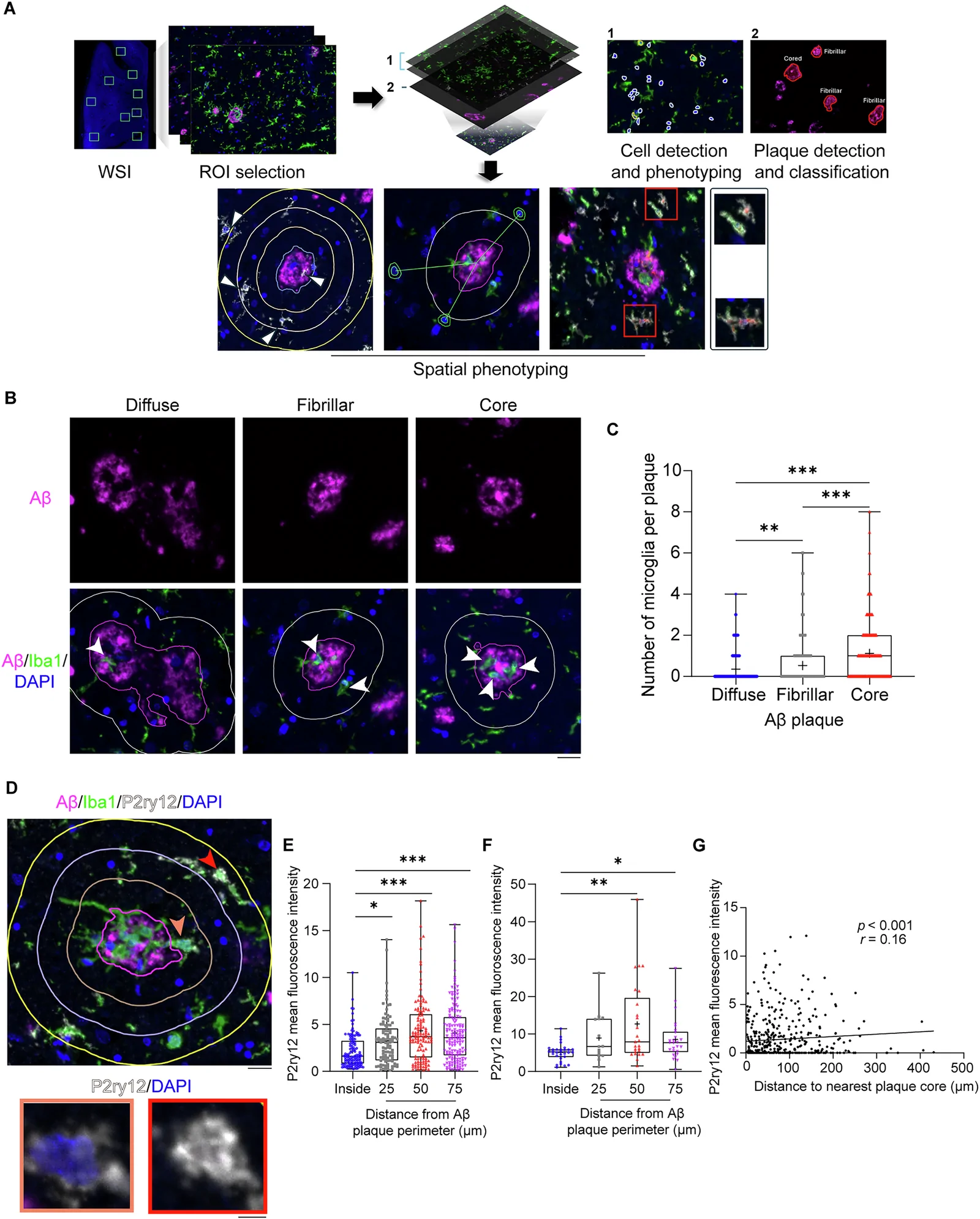
DeepPlaque reveals phenotypic changes of microglia in relation to their distance from different Aβ species. (**A**) Schematic diagram showing the workflow of PlaqueNet-integrated spatial cellular phenotyping from whole-slide imaging (WSI) of postmortem human brain sections. (**B**) Representative multiplex immunofluorescence (mIF) images stained for Iba1 and beta-amyloid (Aβ). Purple and white lines indicate the perimeter of Aβ plaques and the perimeter surrounding an amyloid plaque with a radius extending 25 µm from its center, respectively. Arrowheads indicate Iba1^+^ microglia. Scale bar = 20 µm. (**C**) Quantitative analysis of Aβ plaque-associated microglia (within 25 µm from the Aβ plaque perimeter) in patients with Alzheimer’s disease (AD). *N* = 6; **p = 9.34E-3, ***p = 1E-10 and 1.17E-6; Kruskal–Wallis test. (**D**) Representative mIF images stained for Iba1, P2ry12, and Aβ from sample AD22. Colored lines indicate the perimeter of Aβ plaques (purple) as well as expansion radii of 25 µm (pink), 50 µm (blue), and 75 µm (yellow) from the Aβ plaque perimeter. Arrowheads indicate the P2ry12 staining of two microglia within expansion radii of 75 µm (red) and 25 µm (orange), respectively. Bottom images are magnified versions of the microglia highlighted by arrowheads. Scale bars = 20 μm (top) and 5 µm (bottom). (**E**,** F**) Quantitative analysis of the mean fluorescence intensities (MFIs) of P2ry12 expressed by each microglia with location relative to Aβ plaques for (**E**) AD22 and (**F**) AD8. *p = 0.014, ***p = 1.15E-7, and *p* = 1.26E-7 for (**E**); *p = 0.0495 and **p = 0.0037 for (**F**); Kruskal–Wallis test. (**C**,** E**, **F**) Data shown in box-and-whisker plots include minimum, maximum, and median values as well as 25th and 75th percentiles; plus signs indicate means. (**G**) Correlation analysis of P2ry12 mean fluorescence intensity with the distance of each microglia to its nearest plaque core. *p* = 0.0007, Spearman correlation analysis. Each dot represents one microglia. ROI region of interest. Source data are available online for this figure.

**Figure EV3:**
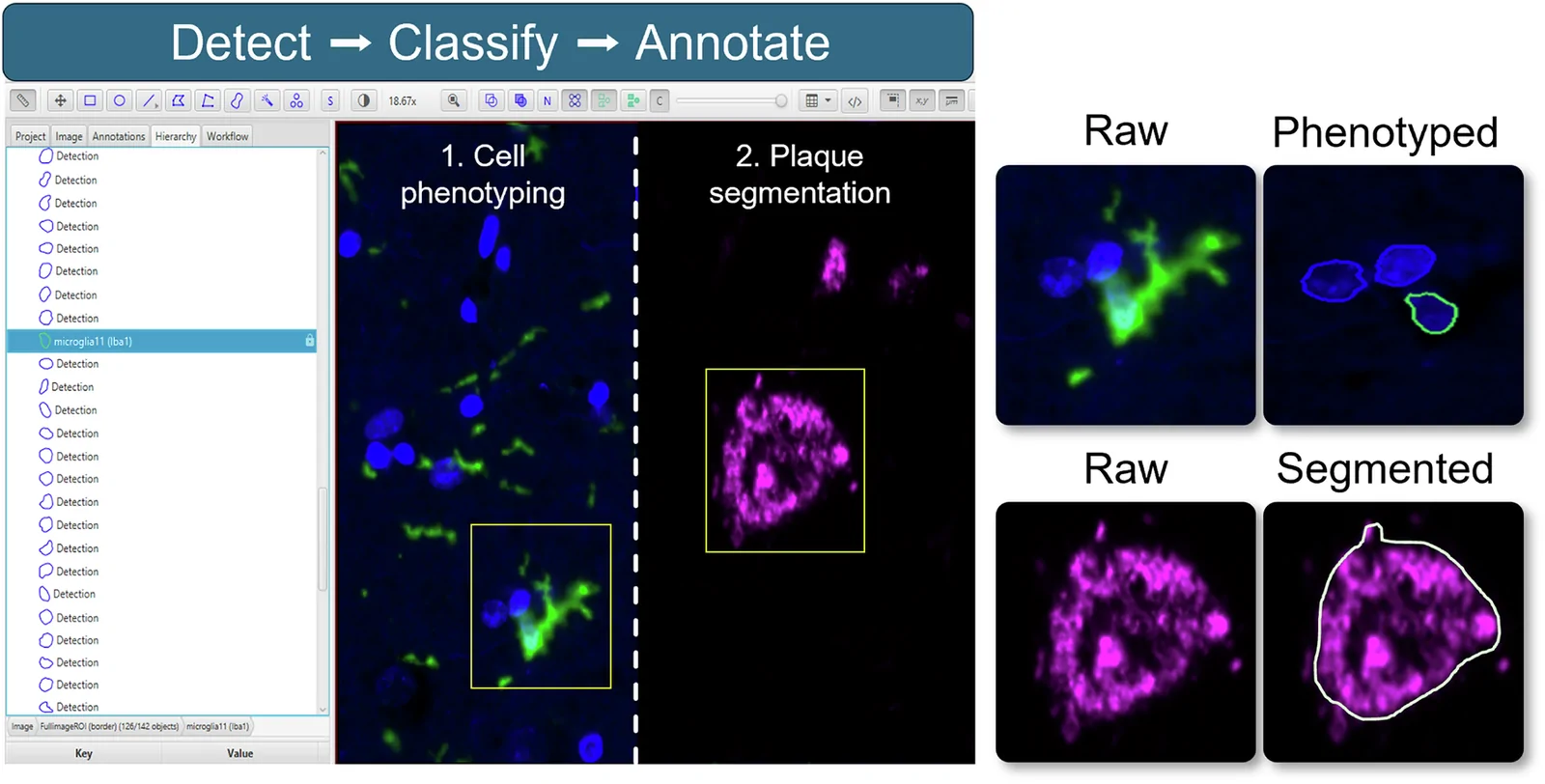
Integration of the DeepPlaque system into QuPath enables automated Aβ plaque detection and spatial cellular phenotyping. Images show representative steps for (1) cell phenotyping of Iba1⁺ microglia (green) and (2) beta-amyloid (Aβ) plaque segmentation (magenta) with both raw and segmented views. Lower-right panels include enlarged images of a fibrillar plaque as raw and segmented images (yellow box).

PlaqueNet enables the detection and classification of distinct Aβ species. However, to investigate the cellular microenvironment around Aβ plaque niches, a scalable and robust spatial cellular phenotyping workflow is pivotal. To minimize manual operation and enhance the robustness of cellular phenotyping for postmortem brain sections, we integrated PlaqueNet and an automated spatial cellular phenotyping workflow based on open-source QuPath software (Bankhead et al, 2017) to analyze multiplex immunofluorescence images (Figs. 3A and EV3). The resultant DeepPlaque system allows customizable spatial cellular phenotyping for detecting and classifying brain cells based on protein marker expression. Here, we demonstrate how we used our DeepPlaque system to examine the Aβ plaque-associated microglia in postmortem brain sections of six AD patients (Figs. 3A and EV3; Dataset EV1).

Accordingly, there were significantly more microglia associated with core Aβ plaques than those associated with both fibrillar and diffuse plaques (both *p* < 0.001; Fig. 3B,C). In addition, more microglia were associated with fibrillar Aβ plaques than diffuse plaques (*p* < 0.01; Fig. 3B,C). This observation aligns with previous reports indicating that more microglia interact with core Aβ plaques than diffuse plaques (Condello et al, 2015; Baik et al, 2016; Spangenberg et al, 2019). We further analyzed the phenotypes of microglia associated with Aβ plaques. Microglia activated in response to Aβ are reported to exhibit decreased expression of the homeostatic microglial marker P2ry12 (Grubman et al, 2021; Keren-Shaul et al, 2017; Mancuso et al, 2024; Sun et al, 2023). Therefore, we used the DeepPlaque system to classify microglia based on their distance from individual Aβ plaques. Notably, microglia located around or inside Aβ plaques exhibited lower levels of P2ry12 than those further away (i.e., 25, 50, or 75 µm from Aβ plaques; *p* < 0.05, *p* < 0.001, and *p* < 0.001, respectively; patient sample AD22; Fig. 3D,E). Another patient sample (AD8) yielded similar findings (Fig. 3F), indicating that microglia in proximity with Aβ plaques exhibit decreased P2ry12 levels. Moreover, the decrease in P2ry12 levels in microglia was correlated with their proximity to Aβ plaques regardless of the heterogeneity of Aβ plaque species (*p* < 0.001; patient sample AD22; Fig. 3G). Similar decreases of homeostatic markers in Aβ plaque-associated microglia have been noted in previous in situ observations of human AD brain tissues (Walker et al, 2020; Kenkhuis et al, 2022; Mancuso et al, 2024). Together, these results indicate that the DeepPlaque system enables the effective examination of microglial phenotypic changes in relation to distinct Aβ plaques.

### DeepPlaque aids locating distinct Aβ species for automated laser microdissection

**Figure 4:**
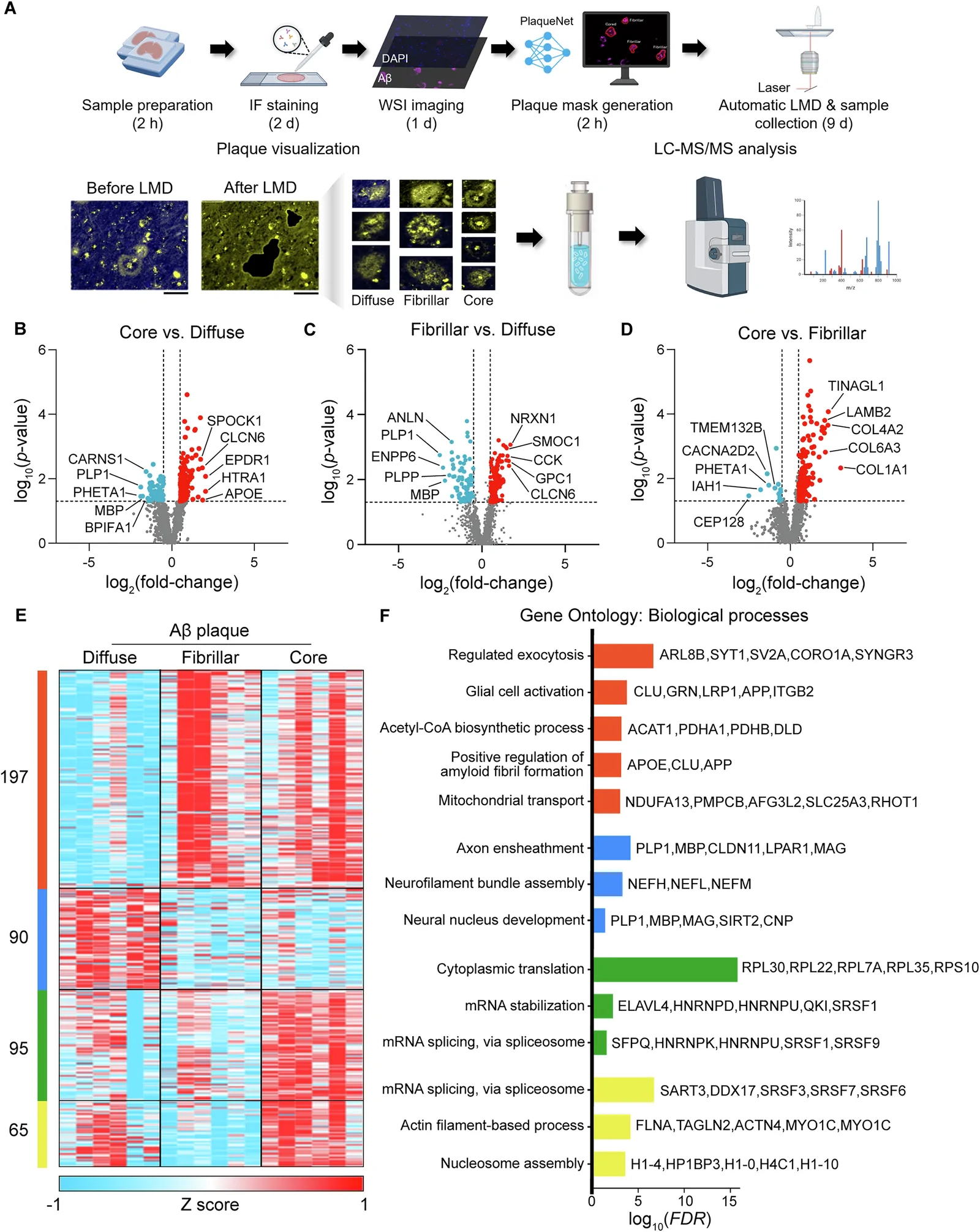
DeepPlaque enables targeted proteomic profiling of Aβ plaque species. (**A**) Workflow of the DeepPlaque system for automated laser microdissection (LMD). Numbers in brackets indicate the time required for each step. Representative images before and after LMD as well as examples of three beta-amyloid (Aβ) species are shown. Aβ plaque species were collected for liquid chromatography–mass spectrometry (LC-MS/MS) analysis. Two biological replicates (i.e., AD22 and AD27) and three technical replicates for each Aβ species were analyzed. Scale bar = 50 µm. Illustrative icons created in BioRender (Jin, C., 2025, https://BioRender.com/mz4nyrx). (**B**–**D**) Volcano plot showing differentially expressed proteins in (**B**) core versus diffuse, (**C**) fibrillar versus diffuse, and (**D**) core versus fibrillar Aβ plaque species. Blue and red dots indicate proteins that are down- or up-regulated, respectively. The top five proteins that are down- or up-regulated are shown. All exact *p* values are listed in Dataset EV2. (**E**,** F**) Heatmap and representative Gene Ontology (GO) terms for biological processes are shown. Four clusters of proteins are grouped based on biological processes. The top three to five upregulated proteins are shown for each protein cluster. The heatmap was generated with Morpheus (https://software.broad-institute.org/morpheus). IF immunofluorescence, WSI whole-slide imaging.

**Figure EV4:**
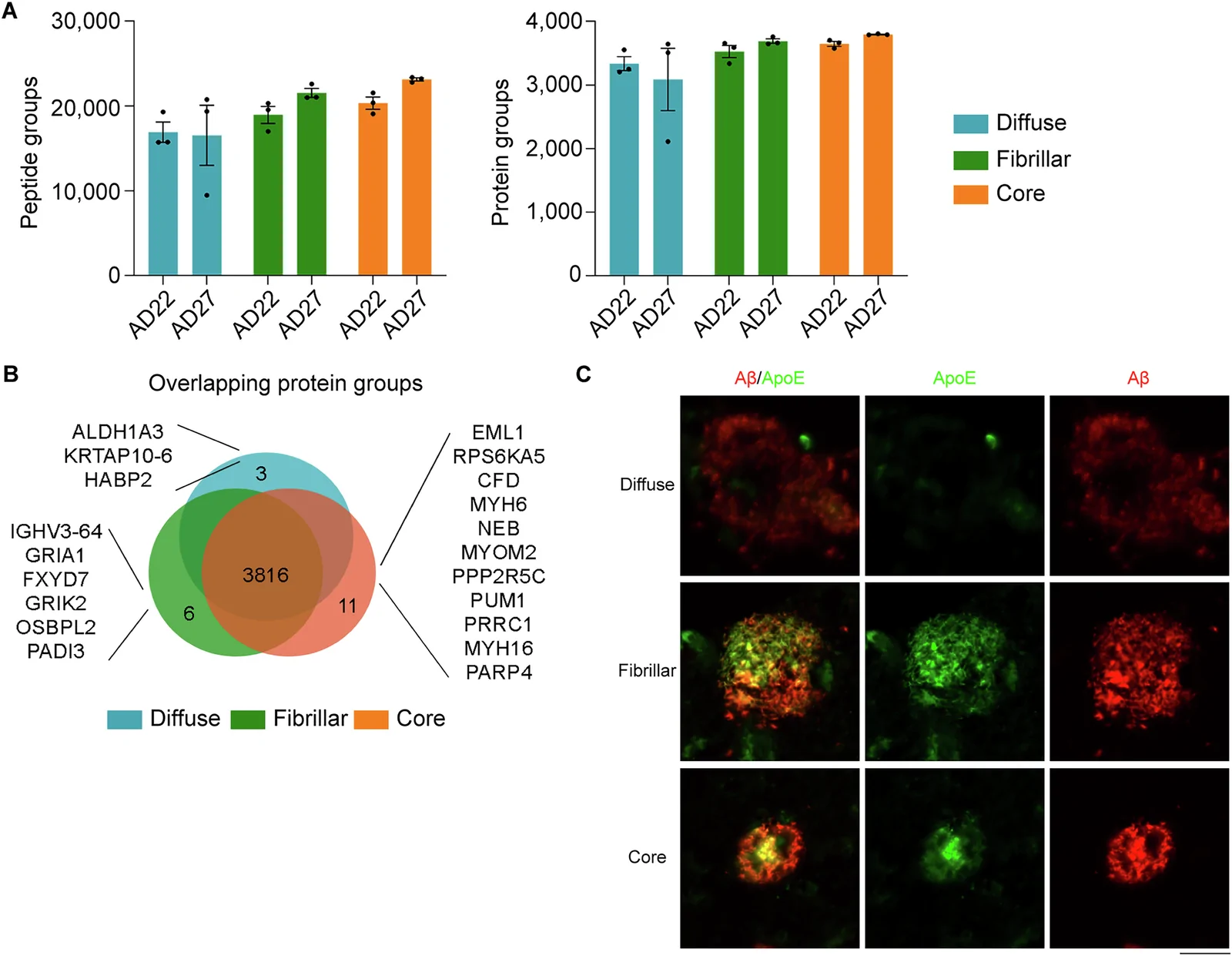
PlaqueNet-assisted targeted laser microdissection of Aβ plaques. (**A**) Peptide and protein groups obtained from postmortem sections of samples AD22 and AD27, serving as two biological replicates. Three technical replicates were analyzed for each type of beta-amyloid (Aβ) plaque. Data shown in histograms include means ± SEM. (**B**) Overlapping proteins between the three types of Aβ plaques. (**C**) Representative images of the three Aβ species stained for Aβ (red) and APOE (green) in AD22. Scale bar = 25 µm.

Understanding the molecular composition of different Aβ plaque species and molecular phenotypes of neighboring cells is crucial for uncovering the biology of Aβ aggregation. As such, we extended the use of PlaqueNet to facilitate spatial proteomics for distinct Aβ plaque species in postmortem human brain sections in a robust, automated, and scalable manner. Using PlaqueNet, we robustly identified and classified three Aβ plaque species with whole-slide images from postmortem brain sections, resulting in a total of 2800 Aβ plaques (Figs. 1B; Dataset EV1). We directly imported the segmentation outputs into automated laser microdissection (LMD) for spatial proteomics (Fig. 4A). We then profiled proteins extracted from the LMD-isolated Aβ plaques by liquid chromatography–tandem mass spectrometry (LC-MS/MS), yielding 3836 proteins across both brain samples (Figs. 4A and EV4A,B). Compared to diffuse Aβ plaques, we identified 222 differentially expressed proteins (DEPs) in fibrillar Aβ plaques and 230 DEPs in core Aβ plaques. In addition, there were 157 DEPs in core Aβ plaques compared to fibrillar Aβ plaques (Fig. 4B–D; Dataset EV2). Concordant with previous findings (Bales et al, 1997; Namba et al, 1991; Ulrich et al, 2018; Boon et al, 2020; Sakono and Zako, 2010; Finder and Glockshuber, 2007), the expressions of APOE (apolipoprotein E) and APP (amyloid precursor protein) were significantly higher in core Aβ plaques than in diffuse Aβ plaques (Fig. 4B; Dataset EV2). We subsequently confirmed the expression of APOE across the three distinct Aβ species using immunofluorescence staining. Accordingly, we observed higher levels of APOE in fibrillar and core Aβ plaques than in diffuse Aβ plaques, suggesting increased colocalization of APOE with specific Aβ plaque species (patient sample AD22; Fig. EV4C).

Interestingly, we found that several proteins reported to be involved in Aβ aggregation, including NRXN1 (neurexin-1), CLCN6 (chloride voltage-gated channel 6), and SMOC1 (SPARC-related modular calcium-binding protein 1), were upregulated in both fibrillar and core Aβ plaques compared to diffuse Aβ plaques (Fig. 4B,C; Dataset EV2). NRXN1, a synaptic protein that plays a role in synapse formation, is enriched in Aβ plaques and may interact with Aβ oligomers (Naito et al, 2017). CLCN6 is suggested to be involved in storing and transporting Aβ, potentially contributing to the formation of Aβ plaques (Martá-Ariza et al, 2025). SMOC1 is colocalized with Aβ plaques and may alter Aβ aggregation and fibrillation (Balcomb et al, 2024). Interestingly, we observed decreased expression of proteins associated with myelination, such as MBP (myelin basic protein) and PLP1 (proteolipid protein 1), in both core and fibrillar Aβ plaques compared to diffuse Aβ plaques (Fig. 4B,C; Dataset EV2). Compared to fibrillar Aβ plaques, core Aβ plaques exhibited differential expression of collagen proteins (i.e., COL1A1, COL4A2, and COL6A3), a glycosylation-related protein (i.e., TINAGL1), and laminin (i.e., LAMB2) (Fig. 4D; Dataset EV2). These extracellular cell adhesion-associated proteins have been reported in proteomic profiles of amyloid plaques in AD brain samples (Liao et al, 2004; Lin et al, 2024; Zhang et al, 2020, 2024; Leitner et al, 2024; Gerrits et al, 2021).

To better understand the molecular changes and biological pathways associated with Aβ plaque development, we compared the proteomic profiles of diffuse, fibrillar, and core Aβ plaque species and performed the Gene Ontology (GO) analysis. Compared to diffuse Aβ plaques, fibrillar and core Aβ plaques had enriched protein pathways of “exocytosis,” “glial cell activation,” and several metabolism-related pathways (e.g., “acetyl-CoA biosynthetic process” and “mitochondrial transport”) (Fig. 4E,F, red cluster), indicating increased glial activity and altered cellular metabolism. Moreover, this cluster contained proteins associated with amyloid fibril formation, including APOE, CLU, and APP. In contrast, fibrillar and core Aβ plaques had fewer proteins related to “axon ensheathment,” suggesting axonal damage (Fig. 4E,F, blue cluster). Furthermore, core Aβ plaques showed upregulated protein pathways related to “cytoplasmic translation,” “mRNA stabilization,” and “mRNA splicing, via spliceosome” (Fig. 4E,F, green and yellow clusters), indicating involvement in transcription regulation. These findings provide a comprehensive proteomic profile of different Aβ plaque species, highlighting their distinct molecular compositions. The proteins enriched in each Aβ plaque species are associated with differential biological pathways, suggesting that altered cellular responses, axonal damage, and transcription regulation contribute to the toxicity and formation of distinct Aβ plaque species.

## Discussion

In this study, we developed the DeepPlaque system, which consists of PlaqueNet—an open-weight deep learning model that accurately detects and classifies diffuse, fibrillar, and core Aβ plaques—along with a workflow for high-resolution automated proteomic profiling of Aβ niches microenvironment. We showed that PlaqueNet outperforms state-of-the-art algorithms and expert researchers while maintaining robustness across different imaging systems. PlaqueNet demonstrates superior accuracy and reproducibility compared to existing methods, addressing a major bottleneck in manual annotation and enabling large-scale, quantitative analysis of Aβ plaque microenvironments, which was previously impractical. Furthermore, through the development and application of the DeepPlaque system, we presented a proof-of-concept study illustrating how this AI-based tool facilitates advanced automated detection and classification of Aβ plaques, enabling spatial cellular phenotyping and proteomic profiling of postmortem AD brain samples in a robust, scalable, and accurate manner.

The compactness of Aβ plaques affects their neurotoxicity as well as glial cell activation and microglial clustering (Yuan et al, 2016; Gotkiewicz et al, 2025; Almeida et al, 2025). Although several studies have investigated the gross composition of Aβ plaques (Drummond et al, 2022; Xiong et al, 2019; Jiao, 2024; Liao et al, 2004), there has been no detailed investigation of the molecular signatures associated with the diffuse, fibrillar, and core Aβ species. Accordingly, the DeepPlaque system enables high-throughput identification of protein signatures associated with 3 specific Aβ species, which is technically challenging to perform manually. The results from volcano plots and GO analysis show that the protein composition of different Aβ species aligns with the current understanding of the biology involved in Aβ plaque formation. While APOE is primarily found in fibrillar and compact Aβ plaques, it is suggested to play key roles in Aβ fibrillation, Aβ plaque compaction, and microglial clustering and activation (Namba et al, 1991; Bales et al, 1997; Holtzman et al, 2000; Ulrich et al, 2018; Boon et al, 2020; Parhizkar et al, 2019). In addition, we observed lower expression of myelination-associated proteins in both core and fibrillar Aβ plaques than in diffuse Aβ plaques. Notably, MBP and its degraded form interact with Aβ peptides in AD brains (Zhan et al, 2015). The loss of myelin-associated proteins from diffuse to fibrillar/core plaques may result from axonal damage caused by the toxic effect of Aβ aggregation. Therefore, understanding the protein composition of different Aβ plaque species is essential for elucidating the biology underlying the formation and evolution of specific Aβ plaque species. Given the growing clinical use of anti-Aβ monoclonal antibodies, the interaction between microglia and distinct Aβ plaque species likely determines the therapeutic outcomes of these treatments (Karran and De Strooper, 2022). Therefore, understanding the regulation of microglia and their interaction with Aβ is important for deciphering therapeutic responses to anti-Aβ monoclonal antibodies and for elucidating the disease mechanisms of AD. For instance, our group reports that factors, such as sex, *APOE* genotype, and genetic variants, are associated with the number of microglia interacting with Aβ plaques (Jiang et al, 2022). Therefore, it would be compelling to leverage our model to further investigate if the increase in amyloid-associated microglia is associated with specific Aβ plaque species. The DeepPlaque system serves as a valuable tool for researchers to conduct standardized and quantitative postmortem analysis. Our spatial cellular phenotyping and detailed proteomic profiling of Aβ plaque niches suggest that DeepPlaque accurately recapitulates known pathology and can effectively profile Aβ plaques with great heterogeneity. In addition, the DeepPlaque system employs immunohistochemical staining with an anti-Aβ antibody, enabling robust multiplex immunostaining of various cell types alongside Aβ pathology. This approach allows for comprehensive cellular characterization beyond mere plaque identification, enabling effective quantification of Aβ pathology and its associated microenvironment.

The robust generalization of PlaqueNet across imaging systems, as demonstrated in transferability tests, supports its use in multicenter studies, addressing a common issue in quantitative neuropathology caused by technical variability (Jahanifar et al, 2025). The spatial cellular phenotyping workflow designed based on QuPath (Bankhead et al, 2017) described in the present study can also be adapted to characterize other cell types, such as astrocytes, neurons, and endothelial cells, as well as diverse molecular phenotypes revealed by different protein marker panels. With the rise of foundation models trained on large-scale biomedical datasets (Vorontsov et al, 2024), the DeepPlaque system could be further integrated with spatial transcriptomics or metabolomics workflows to achieve fully multimodal analyses that link histopathology, molecular signatures, and cellular phenotypes, thereby generating a unified spatial atlas of AD.

Despite the strengths of the DeepPlaque system, this study has several limitations that restrict the scope of our conclusions while remaining consistent with the demonstrated robustness of the system. First, the diversity of available human postmortem tissues suitable for multiplex immunofluorescence and spatial proteomics is inherently limited, partly because of the high cost of high-resolution imaging, the specialized nature of staining protocols, and the adoption of the imaging systems used in this study. Second, although PlaqueNet maintains strong performance under domain shifting and outperforms experienced researchers, we observed an expected reduction in mean average precision on images from an unseen imaging system by PlaqueNet and in the presence of autofluorescence in the staining. Moreover, we excluded morphologically ambiguous plaques from the classification of Aβ plaque species, which may affect mean average precision, specifically if the images processed by PlaqueNet are not of optimal quality. Accordingly, prospective harmonization strategies, such as staining normalization, calibration slides, and domain adaptation, are needed to stabilize the model’s performance across staining protocols and microscope systems. Despite these constraints, the open and modular nature of the DeepPlaque system enables site-specific tuning and protocol adaptation, providing a practical path to addressing these limitations in future multicenter studies.

Building on this foundation, we envision our DeepPlaque system could be further adapted to examine other types of Aβ plaque species, such as neuritic plaques, by incorporating the immunostaining of phosphorylated tau proteins. This adaptability can also extend to exploring other proteinopathies in postmortem brain sections, thereby broadening the system’s utility in neurodegenerative disease research and enhancing our understanding of how amyloid pathology impacts neighboring brain cells. Moreover, as the first proof-of-concept study, our PlaqueNet addresses the classification of the more commonly studied Aβ plaque types—diffuse, fibrillar, and core plaques—which are essential for pathological staging in Alzheimer’s disease research. While the current version focuses on these Aβ plaque types, the underlying architecture has been developed with extensibility in mind, enabling the ready incorporation of additional species, such as neuritic plaques, in future updates. The robustness and accuracy of PlaqueNet demonstrate the power of our neighborhood‑based contrastive strategy, which could be further optimized to examine different types of proteinopathies with specific training databases. On top of the deep learning model, the integrated workflows targeting spatial multi-omics allow automated, large-scale, and in-depth analysis of the pathological hallmarks of neurodegenerative diseases.

## Methods

**Table:** Reagents and tools table

Reagent/resource	Reference or source	Identifier or catalog number
**Experimental models**	**Not applicable**	**Not applicable**
**Recombinant DNA**	**Not applicable**	**Not applicable**
**Antibodies**		
Mouse anti-beta amyloid	Santa Cruz Biotechnology	Cat#sc-32277 Clone NAB228 1:750 dilution
Rabbit anti-Iba1	FUJIFILM Wako Pure Chemical	Cat# 019-19741 Polyclonal 1:500 dilution
Rabbit anti-P2ry12	Novus Biologicals	Cat#NBP233870 Polyclonal 1:200 dilution
Rabbit anti-CD74	Atlas Antibodies	Cat# HPA010592 Polyclonal 1:100
Rabbit anti-HLA-DR	Abcam	Cat# ab257320 Clone HLA-Pan/2967 R 1:100
Rabbit anti-GFAP	Millipore	Cat# AB5804 Polyclonal 1:500 dilution
Rabbit anti-NeuN	Thermo Fisher Scientific	Cat# 702022 Clone 14H6L24 1:300
Mouse anti-CLDN5	Thermo Fisher Scientific	Cat# 35-2500 Clone 4C3C2 1:50
Rabbit anti-ApoE(pan)	Cell Signaling Technology	Cat#13366S Clone D7I9N 1:250
Goat anti-Mouse IgG Alexa Fluor™ Plus 647	Thermo Fisher Scientific	Cat# A32728 1:500
**Oligonucleotides and other sequence-based reagents**	**Not applicable**	**Not applicable**
**Chemicals, enzymes and other reagents**		
Thioflavin-S	Sigma-Aldrich	Cat# T1892
SISPROT PAC Kit	BayOmics Biotechnology	BO-013-24
**Software**		
Phenochart Whole Slide Viewer version 1.1.0	Akoya Biosciences	
inForm Tissue Analysis Software version 2.6.0	Akoya Biosciences	
QuPath version 0.5.0	Bankhead et al, 2017	
Fiji ImageJ version 2.14.0	Schindelin et al, 2012	
Microsoft Power Query version 2.08	Microsoft Corporation	
GraphPad Prism version 9.2.0	GraphPad Software	
TissueFAXS version 7.1.141	TissueGnostics	
StrataQuest version 7.1.1.138	TissueGnostics	
MMI CellCut version 6.1.442	Molecular Machines and Industries	
DL4MMI version 0.1.0	BayOmics Biotechnology	
VistaNavi version 1.1.0	BayOmics Biotechnology	
Spectronaut version 18	Biognosys AG	
**Other**		
Sticky Cap	BayOmics Biotechnology	BO-019-06
50 μm × 20-cm Fused-Silica Capillary Column	BayOmics Biotechnology	RP-210050C18
Vacuum Centrifugal Concentrator	Beijing JM Technology	CV600
timsTOF Pro2	Bruker Daltonics	
EASY-nLC 1000 System	Thermo Fisher Scientific	
Noncontact Ultrasonic Cell Disruptor	Ningbo Scientz Biotechnology	Scientz08-IIIC
MMI Cellcut Laser Microdissection	Molecular Machines and Industries	
MMI MembraneSlides	Molecular Machines and Industries	50103

### Postmortem human brain sections

We obtained postmortem, formalin-fixed, paraffin-embedded, human prefrontal cortex brain sections (7-µm thick) from patients with AD and controls from the MRC UK Brain Banks Network (UKBBN, Bristol Brain Bank), which has approval from the North Somerset and South Bristol Research Ethics Committee to operate as a research tissue bank (REC reference number: 23/SW/0023). Consent was obtained from potential donors while alive, and the capacity to make this decision at the time of registration was witnessed by an appropriate person in a qualifying relationship. If a potential donor no longer had the capacity to consent for themselves, the South West Dementia Brain Bank also accepted applications submitted on their behalf by an appropriate person in a qualifying relationship under specific conditions. The brain bank provides data, including histological diagnosis, age at death, sex, postmortem delay, Braak stage, and age-related plaque scores (Dataset EV3). This study was approved by the Human and Artefacts Research Ethics Committee (HAREC) at the Hong Kong University of Science and Technology (HREP-2025-0644).

### Immunofluorescence staining

#### Multiplex immunofluorescence assay for PlaqueNet development and external testing

We performed manual monoplex and multiplex immunofluorescence staining using an Opal Polaris 7-color Manual IHC kit (Akoya Biosciences, MA, USA), which incorporates tyramide signal amplification-conjugated fluorophores to detect protein marker signals. After deparaffinization and rehydration, we performed epitope retrieval with sodium citrate buffer in a pressure cooker for 20 min. Then, we performed autofluorescence quenching as previously described (Du et al, 2019). We then incubated the sections in 10% goat serum for 30 min followed by primary antibodies for 60 min at room temperature (see Dataset EV4 for a list of antibodies used in this study). After washing, we incubated the sections with anti-mouse and anti-rabbit Opal Polymer HRP (Akoya Biosciences) for 30 min at room temperature. We then treated the sections with the respective Opal fluorophores for 10 min at room temperature. We subsequently placed the sections in the AR6 buffer and began microwave treatment for antibody stripping: we allowed the buffer to boil for 1 min under high-power microwaving at 1100 W, followed by an additional 15 min of low-power microwaving at 100 W with the added sections. We left the sections in buffer to cool to room temperature for 15 min. For monoplex immunofluorescence staining, we performed spectral DAPI (Akoya Biosciences) incubation for 5 min and then mounted the sections with coverslips. For multiplex immunofluorescence staining, we restarted the cycle with 10% goat serum blocking followed by a second round of incubation with primary antibody, secondary antibody, and Opal fluorophore. We continued staining cycles until the final incubation with anti-Aβ antibody (clone NAB228, Santa Cruz Biotechnology, TX, USA), which is commonly used for detecting Aβ in human samples. After the final microwave treatment, we incubated the sections with Opal Polaris 780 for 60 min at room temperature. After washing, we counterstained the sections with spectral DAPI, mounted them with coverslips, and stored them at 4 °C. As a negative control for autofluorescence, we included an unstained section without antibodies or Opal fluorophore. We imaged the sections at 40× magnification with PhenoImager (version 1.0, Akoya Biosciences). Then, we annotated the whole-slide scans using Phenochart Whole Slide Viewer (version 1.1.0, Akoya Biosciences), which we later unmixed and exported as multi-image component TIFF files using inForm Tissue Analysis Software (version 2.6.0, Akoya Biosciences). We randomly selected representative regions of interest (ROIs) from the gray matter of the whole-slide images. To minimize bias in the sampling of Aβ plaques, we enabled only the DAPI channel during random sampling to accurately delineate the gray matter.

#### Transferability test and APOE staining

After deparaffinizing and rehydrating the sections, we performed epitope retrieval with sodium citrate buffer in a pressure cooker for 20 min. Then, we performed autofluorescence quenching as previously described (Du et al, 2019), followed by blocking in 10% goat serum for 60 min. We incubated sections from AD15 and AD19 with 0.2% thioflavin-S (Sigma-Aldrich, MA, USA) in 50% ethanol for 8 min followed by treatment with concentrated PBS in the dark for 10 min at 4 °C as previously described (Sun et al, 2002) with some modifications. After washing, we incubated the sections with anti-Aβ antibody overnight at 4 °C (Dataset EV4). The following day, we washed the tissue sections with Tris-buffered saline with 0.01% Triton X-100 and then incubated them with fluorophore-labeled anti-mouse secondary antibody (Life Technologies, CA, USA) for 60 min at room temperature. After washing, we counterstained sections from AD20, AD21, AD22, and AD23 with DAPI (1:1000 dilution; Invitrogen, MA, USA) and sections from AD15 and AD19 with SYTOX Green Nucleic Acid Stain (1:100,000 dilution; Thermo Fisher Scientific, MA, USA), mounted them with coverslips, and stored them at 4 °C. We captured images using THUNDER Imager (Leica Microsystems, Germany).

### Development of PlaqueNet

#### Generation of image datasets

We quantitatively analyzed annotated images and segmented Aβ plaques based on a fixed pixel threshold using QuPath (version 0.5.0) (Bankhead et al, 2017), in which Aβ plaques are segmented using a consistent pixel intensity threshold. We excluded objects smaller than 300 µm^2^ to focus the analysis on established plaques and algorithmically merged adjacent subthreshold aggregates (i.e., <300 µm^2^, Fig. EV1C) with larger plaques to reflect contiguous pathology. An experienced researcher subsequently classified segmented amyloid plaques as diffuse, fibrillar, or core according to established morphological and signal intensity criteria (Dickson and Vickers, 2001). Diffuse Aβ plaques exhibit low signal intensity with minimal β-sheet fibrillar structure, which is consistent with predominant oligomeric Aβ. Fibrillar Aβ plaques exhibit filamentous Aβ structures. Finally, core Aβ plaques are defined by a dense Aβ core surrounded by a diffuse Aβ halo (Fig. 1C; Dataset EV1). We excluded morphologically ambiguous plaques from classification; images with individual plaques with technical artefacts; images with incomplete Aβ plaques; and images that were of poor quality, out of focus, or overexposed (Fig. EV1C). We exported high-resolution composite TIFF images (1860 ×  1396 pixels, 0.2503 µm per pixel) with Aβ plaques shown in yellow and DAPI shown in blue, together with JSON files generated with the class label and X/Y-coordinates of each Aβ plaque.

For the transferability test dataset, we developed a workflow to generate multiplex immunofluorescence images compatible with our packaged automated algorithm in QuPath (version 0.5.0) (Dataset EV5). This workflow incorporates the *Bio-Formats* importer function (Linkert et al, 2010) in Fiji ImageJ (version 2.14.0) (Schindelin et al, 2012) to export component and composite TIFF images from a raw project or image files acquired by a Leica microscope. We saved component images with individual channels and composite images with RGB-merged channels.

#### Model architecture and implementation detail

We developed PlaqueNet as the core analytical engine of the DeepPlaque system (Fig. EV1A,B). We sampled training patches (1860 × 1396 pixels) from multiplex immunofluorescence whole-slide images, with Aβ plaques provided as the target channel and DAPI as a structural context channel. This two-channel design avoids dependence on experiment-specific protein markers (e.g., Iba1 and P2ry12), ensuring broader applicability across datasets. To increase robustness, both channels underwent extensive data augmentation, including random rotation, flipping, and scaling, before being stacked into the model input tensor.

To initialize PlaqueNet with rich, low-level visual priors, we employed transfer learning from a Swin transformer model pretrained on the ImageNet dataset (Deng et al, 2009), which enables effective feature extraction despite limited domain-specific training data. Building on this backbone, the input image feature then feeds into a region proposal network and multitask heads for simultaneous classification, detection, and segmentation. Its performance is enhanced by two key innovations. The first is a plaque vicinity contrast module that uses InfoNCE-based contrastive loss (${L}_{{\mathrm{con}}}$) to refine feature discrimination by distinguishing a plaque’s feature, embedding ${z}_{i}$ from that of neighboring plaques (i.e., negative samples, ${z}_{k}$) while pulling it closer to its ground-truth match (i.e., positive sample, ${z}_{p}$). The second innovation is a hierarchical task regularization scheme wherein the loss from the classification head guides the more complex detection and segmentation tasks. The total loss function is a weighted sum of the individual task losses—focal loss for classification (${L}_{{\mathrm{cls}}}$), L1 loss for detection (${L}_{\det }$), cross-entropy loss for segmentation (${L}_{{\mathrm{seg}}}$), and contrastive loss (${L}_{{\mathrm{con}}}$)—which can be denoted as the following equation:

1 $$L={{{\rm{\lambda }}}}_{{{\rm{cls}}}}\,{L}_{{{\rm{cls}}}}+{{{\rm{\lambda }}}}_{\det }\left({L}_{\det }+{{\rm{\alpha }}}{L}_{{{\rm{cls}}}}\right)+{{{\rm{\lambda }}}}_{{{\rm{seg}}}}\left({L}_{{{\rm{seg}}}}+{{\rm{\beta }}}\left({L}_{\det }+{{\rm{\alpha }}}{L}_{{{\rm{cls}}}}\right)\right)+{{{\rm{\lambda }}}}_{{{\rm{con}}}}{L}_{{{\rm{con}}}}$$

With the above loss function, training is performed end-to-end for 50 epochs using the AdamW optimizer with a learning rate of 1 × 10^−4^ and a total batch size of 16 implemented in PyTorch (version 2.1.0) with the MMDetection package (version 3.3.0) on 8 NVIDIA 3090 GPUs. Based on empirical validation, we set the loss weights as follows: ${\lambda }_{{\mbox{cls}}}=1.0$, ${\lambda }_{\det }=1.0,\,{\lambda }_{{\mbox{seg}}}=1.0$, and ${\lambda }_{{\mbox{con}}}=0.1$. We set the hierarchical regularization strengths as follows: $\alpha ,\beta =0.2$. To ensure a robust tuning process on our valuable, limited-size, annotated dataset and avoid the potential bias of a single, fixed validation split, we employed a threefold cross-validation strategy using the internal validation set (*N* = 4013 plaques). We selected the set of hyperparameters that yielded the best average performance across the validation folds. We subsequently trained a final PlaqueNet model from scratch on the entire internal validation dataset using the optimal hyperparameters. We then used this single final model for all evaluations on the independent external, transferability, and application test datasets to ensure an unbiased assessment of its generalization performance. For baseline models, we adopted the recommended hyperparameters from their respective original implementations to ensure fair comparison and reproducibility. When comparing PlaqueNet with other leading object detection models, we implemented all models, including YOLOv3, RetinaNet, Faster R-CNN, DETR, and PlaqueNet, using the widely recognized mmdetection open-source toolbox. We strictly adhered to the highly optimized, architecture-specific, default configurations provided by the mmdetection model zoo. These configurations have been extensively tuned by the community to ensure that each specific architecture reaches its maximum potential.

### Application of the DeepPlaque workflow

#### Packaged automated algorithm for spatial cellular phenotyping

To incorporate PlaqueNet’s outputs, we generated a packaged automated algorithm based on Groovy scripting in QuPath (version 0.5.0)(Bankhead et al, 2017) (Figs. 3A and EV3; Dataset EV5). In brief, the algorithm involves the following steps: (1) export of composite TIFF images for PlaqueNet; (2) cell detection and phenotyping of different cell populations based on DAPI and corresponding protein marker mean fluorescence intensities; (3) application of PlaqueNet’s output; (4) determination of the spatial relationships of cell subpopulations with Aβ plaques.

We provide two ways to examine spatial relationships. The first way is to identify cells based on the distance from the Aβ perimeter (i.e., inside Aβ, *X* µm from the Aβ plaque perimeter). The second way is to measure the nearest neighbor distance of each cell from its nearest Aβ plaque core. For each microglia, we identified the nearest Aβ plaque by proximity based on the Euclidean distance between each centroid XY of objects *A* and *B* using the Pythagorean theorem. We created line annotations linking each microglia and its nearest Aβ plaque to generate a spatial map. As some Aβ plaques of variable morphology are not detected by PlaqueNet, we provide a script to detect remaining Aβ plaques by fixed pixel thresholding. We then created a script to remove overlapping Aβ plaques detected by both PlaqueNet and pixel thresholding based on the plaque perimeter boundaries or distances of the perimeter from the plaque centroids. We also developed scripts to export the measurements of annotations (i.e., Aβ plaques and nearest neighbor distance lines) and cells based on their corresponding classification (Dataset EV5). In addition, we formulated commands using Microsoft Power Query (version 2.08, Microsoft Corporation, NM, USA) to combine, append, and merge measurements based on the image file name and PlaqueNet-predicted (i.e., deep learning-predicted) Aβ plaque names (Dataset EV6).

### Section preparation and laser microdissection of Aβ plaques

For section preparation and immunofluorescence staining, we cut 7-µm-thick prefrontal cortical sections from formalin-fixed, paraffin-embedded brain tissue blocks on the designated membrane. After deparaffinization and rehydration, we performed epitope retrieval with sodium citrate buffer for 20 min at 80 °C and let the sections cool in a moist dark chamber for 30 min. Then, we blocked the sections with 10% goat serum for 60 min. After washing, we incubated the sections with mouse anti-Aβ antibody overnight at 4 °C (Dataset EV4). The following day, we washed the tissue sections with Tris-buffered saline with 0.01% Triton X-100 and then incubated them with fluorophore-labeled anti-mouse secondary antibody (Life Technologies) for 60 min at room temperature. After washing, we counterstained the sections with DAPI (1:1000 dilution; Invitrogen), mounted them with coverslips, and stored them at 4 °C.

For image-guided isolation of Aβ plaques, we used the VistaProX platform (BayOmics, China), which is the commercialized version of the Spatial and Cell-type Proteomics (SCPro) platform described previously (Xu et al, 2024) with some modifications. We acquired multiplex whole-slide images at 40× magnification using the digital Pathology Analysis module of VistaProX (Spatially Visualized Proteomics platform, BayOmics). After adjusting the optimal signal intensities, we exported composite whole-slide TIFF images and further processed them using Fiji/ImageJ (version 2.14.0) (Schindelin et al, 2012) to generate multi-crops compatible with PlaqueNet (Dataset EV5). We then analyzed the multi-crops using PlaqueNet and uploaded the resulting JSON files containing Aβ plaque annotations to DL4LMD software (BayOmics) to generate stitched Aβ plaque masks in TIFF format. To facilitate downstream laser-capture alignment, we manually marked the corresponding color-image crops using an image-editing tool by placing a white-filled square with a black border (1250 × 1250 pixels, which corresponds to 0.16 μm per pixel resolution, resulting in a physical dimension of 200 × 200 μm) at a clearly identifiable position on each image. We then imported the marked color images, DL4LMD-generated mask files, and XML files recording the square positions into VistaNavi software (BayOmics). We subsequently loaded the resulting XML files with cutting paths into the laser microdissection module (VistaProX, BayOmics). The imported cutting paths could be further globally adjusted to achieve real-time spatial alignment with the plaque images. We then dissected the target Aβ plaques at 40× magnification in brightfield mode and collected them using a sticky cap.

### Sample preparation for proteomic analysis

We used ion exchange-based protein aggregation capture (iPAC) technology (Xu et al, 2024) with a commercial kit (SISPROT PAC, BayOmics) to prepare protein samples as previously described. Briefly, we transferred the plaques captured on the adhesive caps to 200-μL PCR tubes using a punch needle. Then we added 30 μL lysis buffer in the SISPROT PAC kit to immerse the punched cylinder material with the plaque on it. We extracted the protein by a sonicate–heat–sonicate cycle: we first processed samples with a noncontact sonicator (Scientz08-3C, 450–540 W; 15 s on and 30 s off for 30 cycles for 7 min total sonication time), followed by brief centrifugation for 10 s. We then heated the samples at 90 °C for 90 min (lid temperature: 93 °C), briefly centrifuged them again, and subjected them to one additional sonication cycle.

We subsequently subjected the protein extracts to an iPAC tip for integrated proteomics sample preparation, including protein reduction and alkylation, enzymatic digestion, and peptide desalting, according to the manufacturer’s protocols. We dried the resulting peptides using a vacuum centrifugal concentrator and then dissolved them in 0.1% formic acid with 0.1× iRT (Biognosys, Switzerland) for MS analysis. There were three technical replicates for each type of Aβ plaque and two biological replicates for all types of Aβ plaques (i.e., AD22 and AD27). For each specific plaque type per biological replicate, we pooled the isolated plaques to achieve a total microdissected area of 0.2 mm^2^ (given the 7-µm section thickness).

### Mass spectrometry data acquisition and raw data analysis

We performed liquid chromatography–tandem mass spectrometry (LC-MS/MS) data acquisition on a timsTOF Pro2 instrument (Bruker Daltonik, Germany) coupled with an Easy-nLC 1000 system (Thermo Fisher Scientific) via a CaptiveSpray ion source. We loaded peptides and achieved separation using a 50-μm I.D. × 20-cm fused-silica capillary column (RP-210050C18, BayOmics) at a flow rate of 100 nL/min and column temperature of 50 °C; mobile phase A was 0.1% (v/v) formic acid in water, and mobile phase B was 0.1% (v/v) formic acid in acetonitrile. The gradient program was 80 min as follows: minute 0–50, 2 to 22% B; minute 50–60, 22 to 35% B; minute 60–70, 35 to 80% B; maintained at 80% for 5 min to flush the column, decreased to 2% for the next 3 min, and maintained at 2% for 2 min.

We performed mass spectrometric analysis either in data-independent (diaPASEF) or data-dependent (ddaPASEF) mode. For diaPASEF analysis, 32 isolation windows with *m*/*z* 20 width were defined, covering an *m*/*z* range of 400–1040 with the same 1/*K*_0_ range of 0.75–1.30 V s cm^−2^. We set the collision energy to rise linearly over the covered mobility range from 59 eV at 1/*K*_0_ = 1.6 V s cm^−2^ to 20 eV at 1/*K*_0_ = 0.6 V s cm^−2^. We estimated the total resulting DIA cycle time to be 1.85 s. For ddaPASEF analysis, we scanned precursor ions in the range of *m*/*z* 300–1500 in positive electrospray mode. The 1/*K*_0_ range was 0.75–1.30 V s cm^−2^ with a ramp time of 200 milliseconds and a total cycle time of 1.03 s. Each duty cycle included one MS1 scan and four parallel accumulation-serial fragmentation (PASEF) MS/MS scans. For diaPASEF-acquired MS raw files, we performed a database search using Spectronaut software (version 18, Biognosys) in Hybrid-DIA mode with all parameters set to the BGS Factory Settings (default). We used the ddaPASEF-acquired MS raw files of squares (~1 mm^2^) dissected from plaque sections as the “additional file” for the Hybrid-DIA search; we used the following FASTA files: *Homo sapiens* (UP000005640, reviewed, 20,593 entries) and the contaminant database exported from MaxQuant (version 2.1.4.0). We set protein and peptide false discovery rate (*FDR*) levels to 1% to generate protein identification or quantification data tables.

### Statistical analysis

For the development of the PlaqueNet model, we assessed statistical significance between models using a one-sided Wilcoxon signed-rank test based on 1000 bootstrapped samples (Wilcoxon, 1992) following prior work (Ma et al, 2025). For the application of the PlaqueNet model in spatial cellular phenotyping, we performed the Kruskal–Wallis test to examine the number of microglia and mean fluorescence intensities of microglial P2ry12. We performed Spearman correlation analysis to evaluate the changes in microglial P2ry12 fluorescence intensities with distances from the nearest plaque cores (GraphPad Prism version 9.2.0, GraphPad Software, MA, USA). We filtered out the DEPs using a threshold of log_2_(fold-change) ≤−0.5 or ≥0.5 and an adjusted *p* value less than 0.05. We performed functional annotation of DEPs according to GO analysis (https://geneontology.org) (Carbon et al, 2021; Ashburner et al, 2000).

## Supplementary information

Peer Review File

Dataset EV1

Dataset EV2

Dataset EV3

Dataset EV4

Dataset EV5

Dataset EV6

Source data Fig. 1

Source data Fig. 3

Expanded View Figures

## Data Availability

The mass spectrometry dataset has been deposited into the ProteomeXchange Consortium via the PRIDE (Perez-Riverol et al, 2022) partner repository with the dataset identifier PXD071421. The deposited dataset is available at https://ebi.ac.uk/pride/archive/projects/PXD071421. All DeepPlaque model source code is available under a CC BY-NC-ND 4.0 license at https://www.github.com/ChengJin-git/DeepPlaque. The source data of this paper are collected in the following database record: biostudies:S-SCDT-10_1038-S44321-026-00488-4.
