# Supplementary material for: DeepPlaque: a scalable multimodal platform for Aβ pathology and cell analysis in Alzheimer’s disease
Source: EMBO Mol Med. 2026 Jul 21;18(9):3495–516. doi: 10.1038/s44321-026-00488-4 (PMC13562684; doi:10.1038/s44321-026-00488-4)
Supplement: Supplementary file 1 [file 44321_2026_488_MOESM10_ESM.pdf]

Expanded View Figures

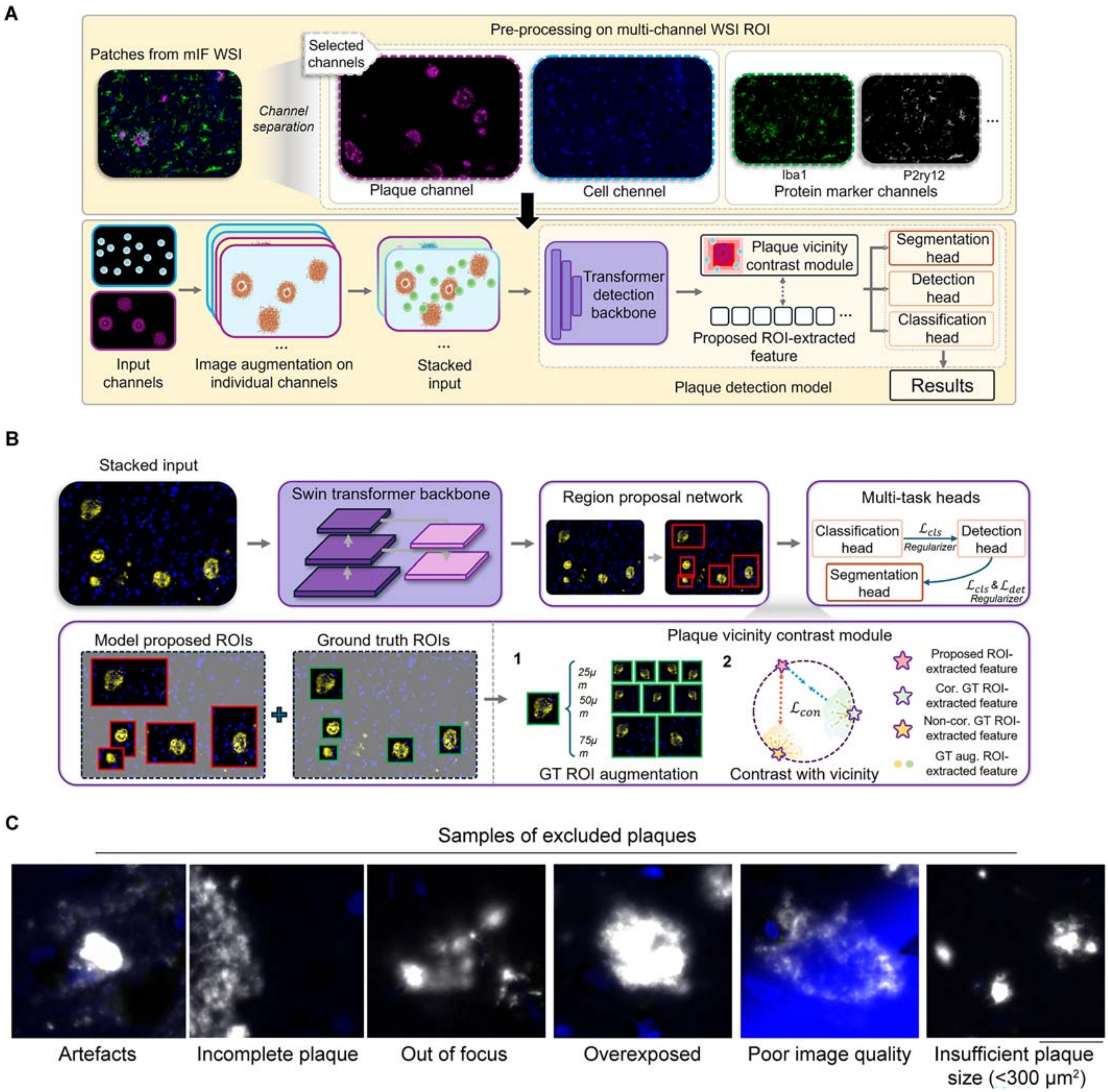

Figure EV1. DeepPlaque preprocessing and PlaqueNet architecture.

(A) Preprocessing of multiplex immunofluorescence (mIF) whole-slide images (WSIs), including channel separation and individual channel augmentations, leading to a stacked input into PlaqueNet. **(B)** PlaqueNet's architecture utilizes a Swin transformer backbone and a region proposal network to feed multitask heads for classification, detection, and segmentation. **(C)** Quality control procedures and illustrations of unsatisfactory plaque samples were excluded. Aug augmented, Cor corrected, GT ground truth, ROI region of interest. Scale bar = 20 μm.

 © The Author(s)

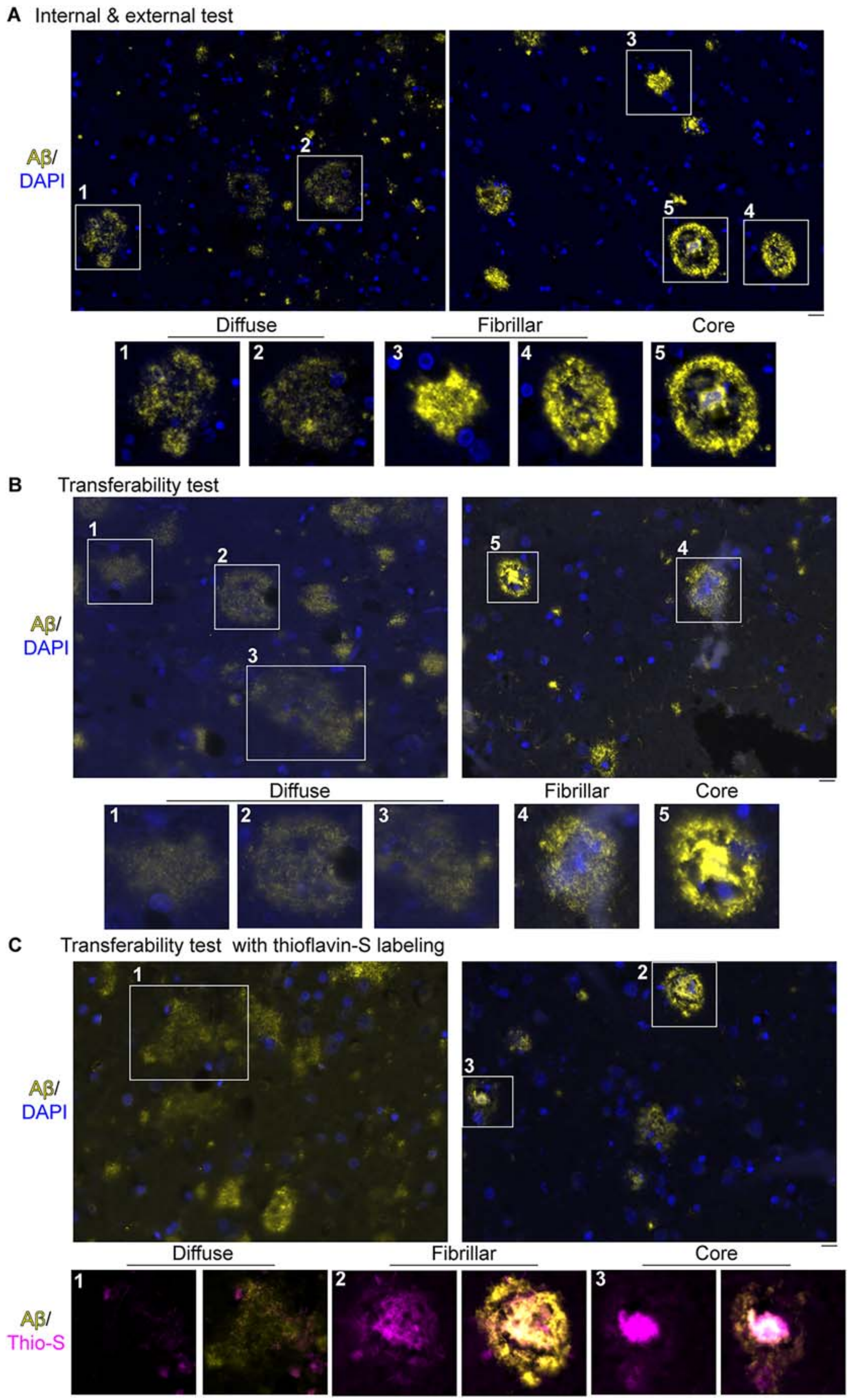
A Internal & external test
Aβ/
DAPI
1
2
3
4
5
Diffuse
Fibrillar
Core
B Transferability test
C Transferability test with thioflavin-S labeling
Thio-S

◀ **Figure EV2. Representative examples of Aβ plaque subtypes detected by PlaqueNet.**

(**A**) Examples from internal and external validation tests stained for beta-amyloid (Aβ, yellow) and nuclei (DAPI, blue). (**B**) Examples of three Aβ plaque species (i.e., diffuse, fibrillar, and core) from transferability testing stained for Aβ (yellow) and nuclei (blue). (**C**) Examples of three Aβ plaque species from transferability testing stained for thioflavin-S (magenta), Aβ (yellow), and nuclei (blue). Scale bar = 20 μm.

 © The Author(s)

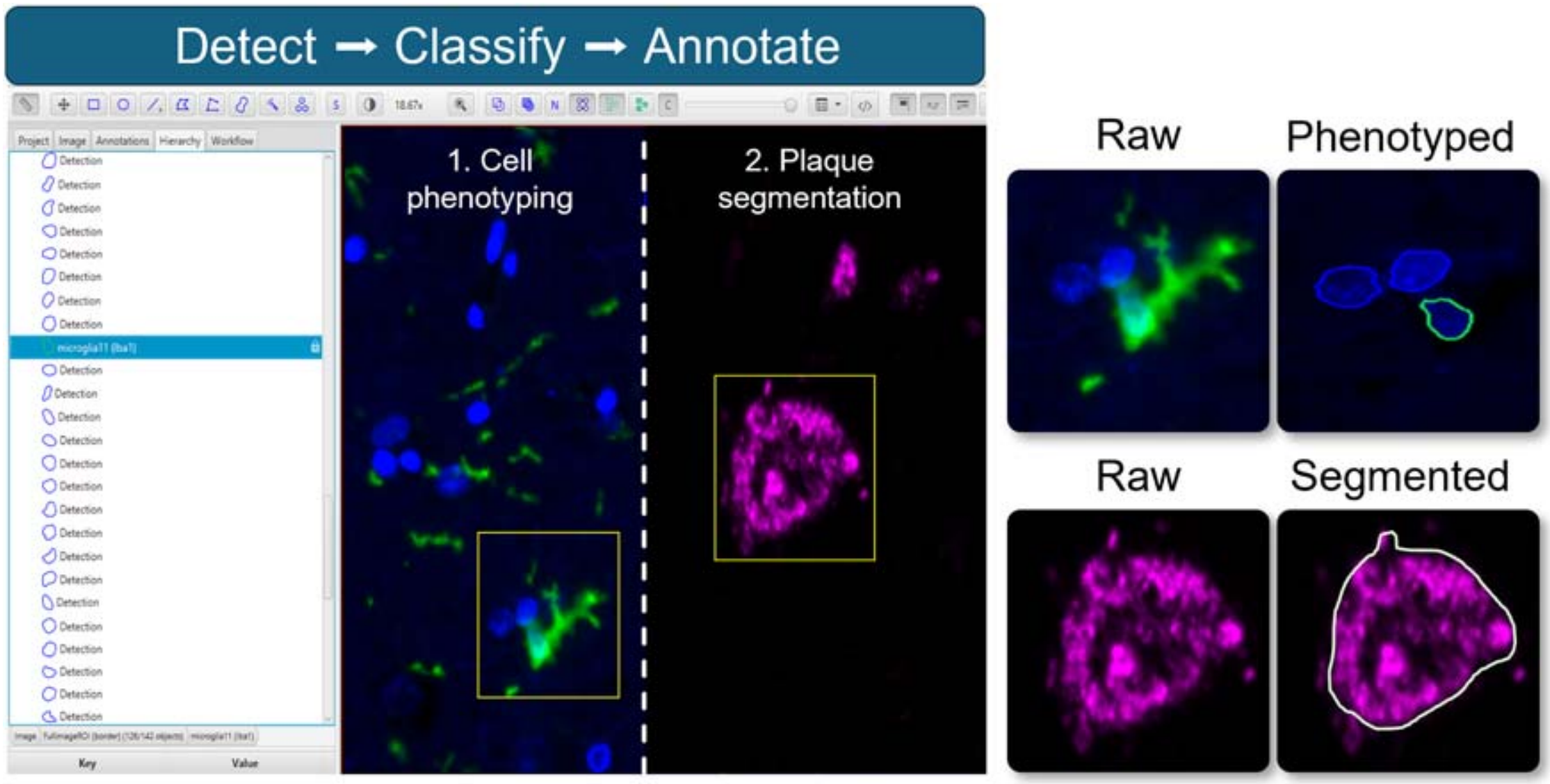

Figure EV3. Integration of the DeepPlaque system into QuPath enables automated Aβ plaque detection and spatial cellular phenotyping.

Images show representative steps for (1) cell phenotyping of $Iba1^+$ microglia (green) and (2) beta-amyloid (Aβ) plaque segmentation (magenta) with both raw and segmented views. Lower-right panels include enlarged images of a fibrillar plaque as raw and segmented images (yellow box).

© The Author(s)

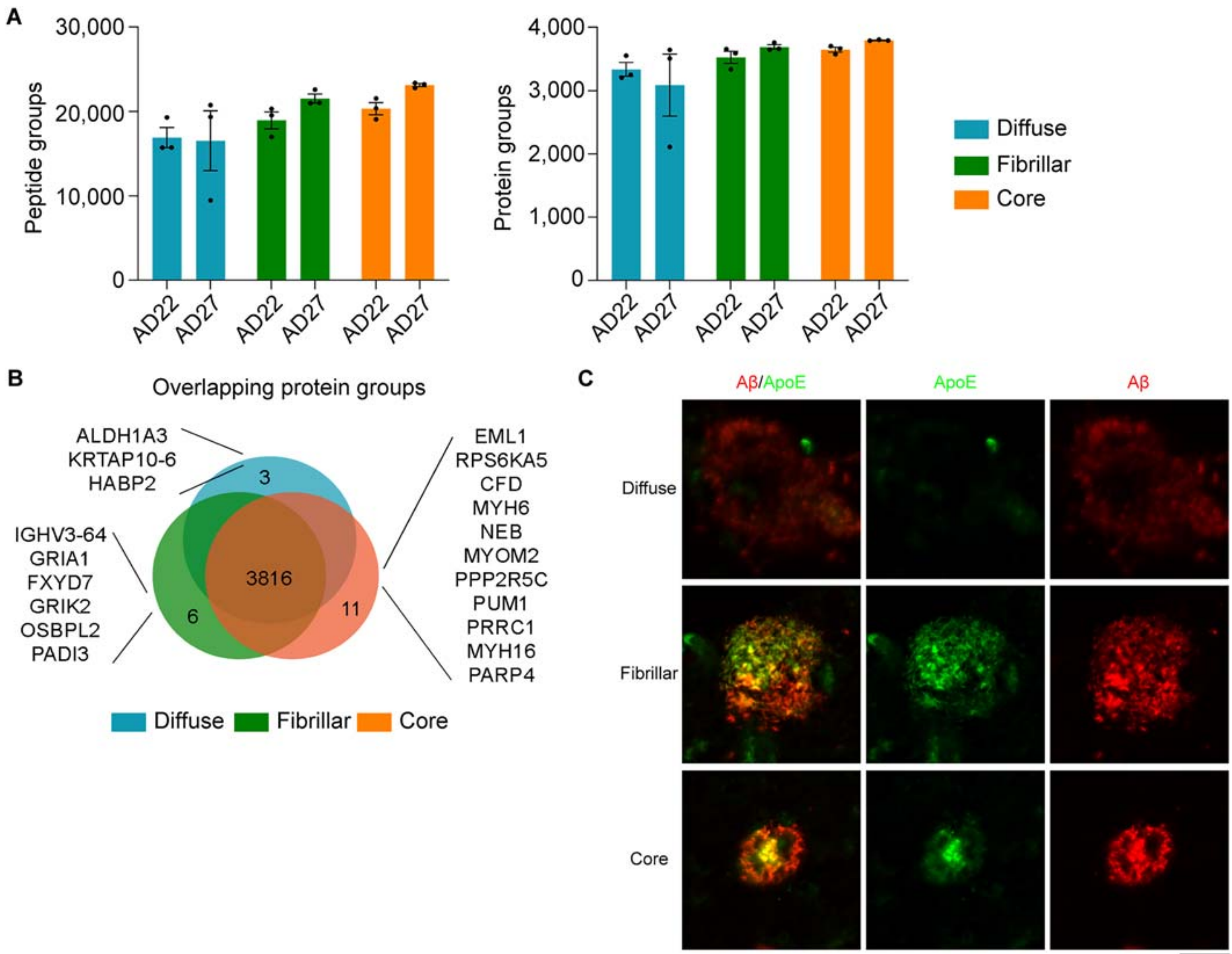

Figure EV4. PlaqueNet-assisted targeted laser microdissection of Aβ plaques.

(**A**) Peptide and protein groups obtained from postmortem sections of samples AD22 and AD27, serving as two biological replicates. Three technical replicates were analyzed for each type of beta-amyloid (Aβ) plaque. Data shown in histograms include means ± SEM. (**B**) Overlapping proteins between the three types of Aβ plaques. (**C**) Representative images of the three Aβ species stained for Aβ (red) and APOE (green) in AD22. Scale bar = 25 μm.

 © The Author(s)
