# Supplementary material for: DeepPlaque: a scalable multimodal platform for Aβ pathology and cell analysis in Alzheimer’s disease
Source: EMBO Mol Med. 2026 Jul 21;18(9):3495–516. doi: 10.1038/s44321-026-00488-4 (PMC13562684; doi:10.1038/s44321-026-00488-4)
Supplement: Supplementary file 2 [file 44321_2026_488_MOESM1_ESM.pdf]

EMBO Molecular Medicine

DeepPlaque: A scalable multimodal platform for Aβ pathology and cell analysis in Alzheimer's disease

Hiu Wong, Cheng Jin, Sze Yuen, Xin Yang, Sunveer Gill, Jiahui Xu, Kuo Lai, Wing-Yu Fu, Weina Gao, Xi Wang, Jiani Wang, Han Cao, Ge Lv, Kin Mok, Ruijun Tian, Amy Fu, Hao Chen, and Nancy Ip

Corresponding authors: Nancy Ip (boip@ust.hk) , Hao Chen (jhc@ust.hk)

Review Timeline:

Submission Date:	6th Mar 26
Editorial Decision:	9th Apr 26
Revision Received:	19th May 26
Editorial Decision:	10th Jun 26
Revision Received:	5th Jul 26
Accepted:	6th Jul 26

Editor: Jingyi Hou

Transaction Report:

(Note: With the exception of the correction of typographical or spelling errors that could be a source of ambiguity, letters and reports are not edited. Depending on transfer agreements, referee reports obtained elsewhere may or may not be included in this compilation. Referee reports are anonymous unless the Referee chooses to sign their reports.)

9th Apr 2026

Dear Prof. Ip,

Thank you again for submitting your work to EMBO Molecular Medicine. We have now received feedback from the three referees who agreed to evaluate your manuscript. As you will see in the reports below, Referee #1 raised some concerns about the study's overall novelty and level of advancement, whereas Referees #2 and #3 were generally supportive. Taking all three reports into account, we have decided to invite a revision.

The referees' recommendations are relatively clear, so I will not repeat them here. In revising the manuscript, it is important to provide the missing details, carefully address the technical questions raised, and more clearly position the work within the existing literature. In particular, the distinctions from prior work and the specific novel contributions of your approach should be articulated more explicitly, and any overstatements should be avoided.

As you may already know, our editorial policy allows in principle a single round of major revision, and it is therefore essential to provide responses to the referees' comments that are as complete as possible. Please feel free to contact me in case you would like to discuss in further detail any of the issues raised by the referees.

EMBO Molecular Medicine has a "scooping protection" policy, whereby similar findings that are published by others during review or revision are not a criterion for rejection. Should you decide to submit a revised version, I do ask that you get in touch after three months if you have not completed it, to update us on the status.

Please also contact us as soon as possible if similar work is published elsewhere. If other work is published, we may not be able to extend the revision period beyond three months.

Please read below for important editorial formatting and consult our author's guidelines for proper formatting of your revised article for EMBO Molecular Medicine.

I look forward to receiving your revised manuscript.

Kind regards,
Jingyi

Jingyi Hou
Senior Editor
EMBO Molecular Medicine

*****
When preparing your revised manuscript, please refer to our guidelines: https://link.springer.com/journal/44321/submission-guidelines#cms-Revised-submissions. We perform an initial quality control of all revised manuscripts before re-review; failure to include requested items will delay the evaluation of your revision.

We require:

1) A .docx formatted version of the manuscript text (including legends for main figures, EV figures and tables). Please make sure that the changes are highlighted to be clearly visible.

2) Individual production quality figure files as .eps, .tif, .jpg (one file per figure). For guidance, download the 'Figure Guide PDF': https://media.springernature.com/original/springer-cms/rest/v1/content/27825798/data/v1.

3) A .docx formatted letter INCLUDING the reviewers' reports and your detailed point-by-point responses to their comments. As part of the EMBO Press transparent editorial process, the point-by-point response is part of the Review Process File (RPF), which will be published alongside your paper.

4) A complete author checklist, which you can download from our author guidelines. Please insert information in the checklist that is also reflected in the manuscript. The completed author checklist will also be part of the RPF.

5) Please note that all corresponding authors are required to supply an ORCID ID for their name upon submission of a revised manuscript.

6) It is mandatory to include a 'Data Availability' section after the Materials and Methods. Before submitting your revision, primary datasets produced in this study need to be deposited in an appropriate public database, and the accession numbers and database listed under 'Data Availability'. Please remember to provide a reviewer password if the datasets are not yet public.

In case you have no data that requires deposition in a public database, please state so in this section. Note that the Data Availability Section is restricted to new primary data that are part of this study.

7) For data quantification: please specify the name of the statistical test used to generate error bars and P values, the number (n) of independent experiments (specify technical or biological replicates) underlying each data point and the test used to calculate p-values in each figure legend. The figure legends should contain a basic description of n, P and the test applied. Graphs must include a description of the bars and the error bars (s.d., s.e.m.).

8) At EMBO Press we ask authors to provide source data for the main manuscript figures. You will receive a separate email with instructions for providing source data with your revised manuscript, including how to upload and organize the files.

9) Our journal encourages inclusion of *data citations in the reference list* to directly cite datasets that were re-used and obtained from public databases. Data citations in the article text are distinct from normal bibliographical citations and should directly link to the database records from which the data can be accessed. In the main text, data citations are formatted as follows: "Data ref: Smith et al, 2001" or "Data ref: NCBI Sequence Read Archive PRJNA342805, 2017". In the Reference list, data citations must be labeled with "[DATASET]". A data reference must provide the database name, accession number/identifiers and a resolvable link to the landing page from which the data can be accessed at the end of the reference.

10) We replaced Supplementary Information with Expanded View (EV) Figures and Tables that are collapsible/expandable online. A maximum of 5 EV Figures can be typeset. EV Figures should be cited as 'Figure EV1, Figure EV2" etc... in the text and their respective legends should be included in the main text after the legends of regular figures.

- For the figures that you do NOT wish to display as Expanded View figures, they should be bundled together with their legends in a single PDF file called *Appendix*, which should start with a short Table of Content. Appendix figures should be referred to in the main text as: "Appendix Figure S1, Appendix Figure S2" etc.

- Additional Tables/Datasets should be labeled and referred to as Table EV1, Dataset EV1, etc. Legends have to be provided in a separate tab in case of .xls files. Alternatively, the legend can be supplied as a separate text file (README) and zipped together with the Table/Dataset file.

11) The paper explained: EMBO Molecular Medicine articles are accompanied by a summary of the articles to emphasize the major findings in the paper and their medical implications for the non-specialist reader. Please provide a draft summary of your article highlighting

- the medical issue you are addressing,

- the results obtained and

- their clinical impact.

This may be edited to ensure that readers understand the significance and context of the research. Please refer to any of our published articles for an example.

12) Author contributions: You will be asked to provide CRediT (Contributor Role Taxonomy) terms in the submission system. These replace a narrative author contribution section in the manuscript.

13) A Conflict of Interest statement should be provided in the main text.

14) Please provide a 'Synopsis' to further enhance discoverability. Synopses are displayed on the journal webpage and are freely accessible to all readers. They include a short stand first (maximum of 300 characters, including space) as well as 2-5 one-sentences bullet points that summarizes the paper. Please write the bullet points to summarize the key NEW findings. They should be designed to be complementary to the abstract - i.e. not repeat the same text. We encourage inclusion of key acronyms and quantitative information (maximum of 30 words / bullet point). Please use the passive voice. Please attach these in a separate file or send them by email, we will incorporate them accordingly.

Please also provide a visual abstract to illustrate your article as a PNG file 550 px wide x 300 px high.

15) All Materials and Methods need to be described in the main text using our 'Structured Methods' format. According to this format, the Methods section includes a Reagents and Tools Table (listing key reagents, experimental models, software and relevant equipment and including their sources and relevant identifiers) followed by a Methods and Protocols section describing the methods, ideally using a step-by-step protocol format. The aim is to facilitate adoption of the methodologies across labs.

Please download and fill our Reagents and Tools Table template (.docx), which you can find in our author guidelines: https://link.springer.com/journal/44320/submission-guidelines#structuredmethods.

When submitting your revised manuscript, please do not include the Reagents and Tools Table in the Methods section of the manuscript but upload it as a separate file choosing the file type "Reagent Table".

EMBO Molecular Medicine has a "scooping protection" policy, whereby similar findings that are published by others during review or revision are not a criterion for rejection. Should you decide to submit a revised version, I do ask that you get in touch after three months if you have not completed it, to update us on the status.

***** Reviewer's comments *****

Referee #1 (Remarks for Author):

Wong et al describe a deep learning approach to classify plaque Abeta morphotypes based on fluorescent IHC stainings to guide subsequent laser microdissection and proteomics.

1) The study, while technically interesting lacks novelty with respect to conceptual and technical innovation. For instance, a similar approach has been presented by Koutarapu et al 2025 (PMID: 40274785), interfacing IF microscopy and deep learning for plaque classification.
With respect to this previous data the current approach lacks biological detail such as characterizing plaques towards Tau/dystrophic neurites (neuritic plaques). Previous studies, as the one above, also used structure sensitive amyloid probes to characterize plaque polymorphs which is superior towards sole IHC. The authors mention that plaques were characterized based on Abeta sheet content but no such methods or data evaluating this are presented.

2) The current study does focus on diffuse, fibrillar and cored plaques without providing any unbiased readout on how these plaques were classified. This is highlighted in the absence of e.g. coarse grain plaques (Boon et al, PMID: 32926214) or any further distinction of senile plaques into cored without neuritic content and neuritic plaques.

3) Lastly, the proteomics has been performed on an unknown number of plaques/plaque type from solely two patients. In general, new proteomics tools allow single cell analysis. Consequently, only a low number of plaques should be needed for sufficient signal. This is troublesome given pathological heterogeneity and intra patient variation.

Referee #2 (Comments on Novelty/Model System for Author):

In the manuscript ""DeepPlaque: A scalable multimodal platform for Aβ pathology and brain cell analysis in Alzheimer's" a pipeline was developed to detect and classify Aβ plaques and cells in immunofluorescence images and combine the analysis with other modalities like spatial proteomics. The idea is relevant, the approach is appropriate, the code was published with annotations, and the conclusions seem realistic. The presented application examples of microglia analysis and proteomics consist of only on two cases but the results appear relatively meaningful.

Referee #2 (Remarks for Author):

In the manuscript "DeepPlaque: A scalable multimodal platform for Aβ pathology and brain cell analysis in Alzheimer's", the authors developed a pipeline to integrate Amyloid beta and cell image analysis with spatial protein dissection. In detail, the model PlaqueNet was created to classify Amyloid beta plaques in immunofluorescence stains of postmortem human brains from Alzheimer's disease patients into three classes: diffuse, fibrillar, and core plaques. Additionally, microglia cells were automatically detected in six cases. Proteins within the plaques were further analyzed by automated laser microdissection and liquid chromatography-tandem mass spectrometry in two cases. The plaque classification model achieved high performance scores. Microglia were more associated with cored and fibrillar plaques than with diffuse plaques. Integrated protein analysis suggested a high ApoE accumulation and an axonal damage within cored plaques. The methods and results are mostly described in detail and in a comprehensible manner. The code was made publicly available. The following comments might help to improve and the manuscript:

1. How does PlaqueNet react with cerebral amyloid angiopathy (CAA) as input?
2. Did you try to train a plaque classification model without the DAPI channel? If yes, did it perform differently?
3. The fact "We excluded morphologically ambiguous plaques from classification" should be somehow mentioned in the limitations as this might cause challenges for users when the model is applied on real (messy) data.
4. Can you add additional information on how the alternative models (YOLOv3, RetinaNet,...) were optimized to get a better impression of how fair the model comparison was?
5. Despite a small n=6, is there a detectable effect of age, sex or ApoE genotype on the number of microglia per plaque (Fig. 3c)?
6. If there is space left, the most relevant results of the microglia and protein analysis might be added in the abstract to convey to readers that these content-related sections are also presented in the manuscript besides the methodology.
7. Fig. 2a: Why is the mean average precision (mAP) of the model 51.7 %? Can you explain a bit more how the score is calculated and what it practically means to have a mAP of 51.7 % when a user applies the model?
8. Fig. 3a (left top image): How were the representative regions from whole slide images selected?
9. If not avoided because of a specific reason, the word "disease" might be added in the title: "DeepPlaque: A scalable multimodal platform for Aβ pathology and brain cell analysis in Alzheimer's disease"
10. Extended Fig. 3: A note in the caption to which class the Abeta plaque belongs to would be convenient.
11. According to the Data Availability statement "The mass spectrometry dataset is deposited to the ProteomeXchange Consortium via the PRIDE (69) partner repository with the dataset identifier PXD071421.". However, I was not able to find the dataset. Can you double check the identifier and add further information so that it is easier to find it?

Referee #3 (Comments on Novelty/Model System for Author):

The authors describe an amyloid plaque classification tool that can be used to automate classification and selection of plaques for laser microdissection. This has the potential to be a useful tool to accelerate local amyloid assessments and heterogeneity assessments.

Referee #3 (Remarks for Author):

This study describes a new tool to automate the classification of amyloid plaques and to integrate this classification with selection of plaques for laser capture microdissection to perform MS of local plaque niches. The authors use three classifications: diffuse, fibrillar, and core. The PlaqueNet algorithm exhibited excellent ability to accurately quantify plaques compared to expert humans. When coupled with glial staining and spatial proteomics, the DeepPlaque system was able to identify differences in plaque niches consistent with previous observations (ie differential APOE or homeostatic microglia markers). Overall, this is a promising tool. The study could be strenghtened by a more diverse classification of amyloid heterogeneity, more robust histological staining, and a more thorough description of methods.

Major critiques:
1.) The antibodies used to assess different pathologies are not described in the methods.
2.) The classification of Abeta staining into 3 groups may limit the utility of the tool. For one, the description of a plaque as "diffuse" without the use of a thioflavin-based dye may reduce accuracy. Second, other forms of amyloid pathology such as coarse-grained (PMID: 32926214), or cotton wool (PMID: 11706935) could be considered.
3.) The histological applicability of DeepPlaque is assessed using only P2ry12 staining. Additional approaches could be used to assess the accuracy and utility of DeepPlaque, such as staining for neuritic plaque-tau, which should be absent in diffuse plaques, or a typical DAM marker to show relative increase around fibrillar plaque.

Re: EMM-2026-23599-T

Title: DeepPlaque: A scalable multimodal platform for Aβ pathology and cell analysis in Alzheimer's disease

Point-by-point responses to reviewers:

Referee #1 (Remarks for Author):

Wong et al describe a deep learning approach to classify plaque Abeta morphotypes based on fluorescent IHC stainings to guide subsequent laser microdissection and proteomics.

1) The study, while technically interesting lacks novelty with respect to conceptual and technical innovation. For instance, a similar approach has been presented by Koutarapu et al 2025 (PMID: 40274785), interfacing IF microscopy and deep learning for plaque classification.

With respect to this previous data the current approach lacks biological detail such as characterizing plaques towards Tau/dystrophic neurites (neuritic plaques). Previous studies, as the one above, also used structure sensitive amyloid probes to characterize plaque polymorphs which is superior towards sole IHC. The authors mention that plaques were characterized based on Abeta sheet content but no such methods or data evaluating this are presented.

We appreciate the reviewer's thoughtful comments and insights. Both our study and that of Koutarapu et al. (2025, PMID: 40274785) developed tools utilizing deep learning models to identify various types of Aβ plaques; however, we would like to emphasize the significant differences in our core concepts and methodologies.

Koutarapu et al. developed a deep learning model to analyze Aβ plaque species based on staining with 2 luminescent, conjugated, thiophene fluorophores obtained from fluorescence microscopy combined with MALDI techniques, focusing on Aβ plaque polymorphism and composition. In contrast, in our study, we developed a deep learning model (i.e., PlaqueNet) to classify different Aβ plaque species from images acquired through immunohistochemical staining using an anti-Aβ antibody with fluorescence microscopy. Our AI platform, PlaqueNet, enables us to analyze Aβ plaque pathology from various imaging platforms, as demonstrated in the external and transferability tests. Immunohistochemical staining with anti-Aβ antibody is widely accepted for detecting Aβ pathology in both research and pathological contexts, and can be integrated with multiplex staining for further studies. In addition, PlaqueNet is equipped with user-friendly FIJI-based scripts to facilitate image export and processing, enhancing its applicability across laboratories with different fluorescence microscope settings.

Accordingly, we added the following sentences to the description in the original manuscript to discuss the limitations of existing AI-based analyses in the *Introduction* (page 4, lines 89-95):

> *“This facilitates high-throughput analysis, disease staging, and the study of Aβ plaque polymorphism and composition (19–22). Although these methods can effectively estimate global Aβ plaque burden, they often rely on coarse summary metrics and overlook the heterogeneity of Aβ plaque species, which exhibit diverse morphologies, variable compaction, and poorly defined boundaries. Moreover, most of these deep learning models have limited generalizability across immunostaining protocols and imaging systems.“*

Beyond the development of our deep learning model, PlaqueNet, our study offers conceptual novelty through a scalable platform that integrates deep learning-based Aβ plaque classification with an automated workflow for spatial histology and proteomics related to Aβ pathology. Our DeepPlaque system employs immunohistochemical staining with an anti-Aβ antibody, enabling robust multiplex immunostaining of various cell types alongside Aβ pathology. This approach facilitates comprehensive cellular characterization beyond mere plaque identification, allowing for effective quantification of Aβ pathology and its associated microenvironment. Our study serves as a proof-of-concept that illustrates how our AI-based tool enhances advanced automated detection and classification of Aβ plaques, enabling spatial cellular phenotyping and proteomic profiling of postmortem brain samples in a robust, scalable, and accurate manner.

Accordingly, we added the following sentences to highlight the conceptual novelty in the *Discussion* section (page 11, line 324–329):

> *“In addition, the DeepPlaque system employs immunohistochemical staining with an anti-Aβ antibody, enabling robust multiplex immunostaining of various cell types alongside Aβ pathology. This approach allows for comprehensive cellular characterization beyond mere plaque identification, enabling effective quantification of Aβ pathology and its associated microenvironment.”*

In addition, PlaqueNet demonstrates algorithmic innovation and technical novelty. PlaqueNet uses a context-aware transformer, which is a structural advancement designed with biological spatial priors. Operating on massive tissue fields (1,860 × 1,396 pixels), we utilized a Swin transformer backbone equipped with a novel plaque vicinity contrast module. By employing InfoNCE-based contrastive learning, PlaqueNet explicitly learns the macroscopic spatial relationships of an Aβ plaque relative to its complex microenvironment. PlaqueNet can distinguish an Aβ plaque’s embedding from its neighboring negative background (see *Model architecture and implementation detail*; page 16, line 480–482, and 485–489). This hierarchical, multitask architecture incorporates simultaneous Aβ plaque classification, detection, and segmentation, going far beyond pure convolutional neural network (CNN)-based local morphological feature extraction.

2) The current study does focus on diffuse, fibrillar and cored plaques without providing any unbiased readout on how these plaques were classified. This is highlighted in the absence of e.g. coarse grain plaques (Boon et al, PMID: 32926214) or any further distinction of senile plaques into cored without neuritic content and neuritic plaques.

We apologize for the lack of clarity. In the original manuscript, we included the definition of the 3 Aβ plaque types (*Generation of image datasets*; page 15, line 452–456) and examples of these 3 Aβ plaque types in Fig. 1C. Our study specifically addresses the classification of the more commonly studied Aβ plaque types—diffuse, fibrillar, and core plaques—which are essential for pathological staging in Alzheimer's disease research in accordance with established criteria (Dickson et al., PMID: 11483304; Tang et al., PMID: 31092819). Moreover, our cohort of postmortem brain samples predominantly includes late-onset Alzheimer's disease cases, which exhibit these 3 major Aβ plaque species.

As noted in our response to Question 1 above, our study serves as a proof-of-concept to illustrate the integration of a deep learning model with an automated workflow for Aβ plaque species and spatial proteomic profiling of postmortem brain samples in Alzheimer's disease. We agree that establishing the foundation of the current DeepPlaque system opens the pathway for advancements in detecting and classifying additional Aβ plaque species, including neuritic plaques. However, this endeavor would require the staining for p-tau and retraining the model. We hope the reviewer agrees that this could be the focus of future studies. Accordingly, we added the following to the *Discussion* (page 12, line 359–362):

> *"While the DeepPlaque system mainly focuses on Aβ plaque species and surrounding cells, we envision its potential to be adapted to examine other types of Aβ plaque species, such as neuritic plaques, as well as other types of proteinopathies in postmortem brain sections."*

3) Lastly, the proteomics has been performed on an unknown number of plaques/plaque type from solely two patients. In general, new proteomics tools allow single cell analysis. Consequently, only a low number of plaques should be needed for sufficient signal. This is troublesome given pathological heterogeneity and intra patient variation.

We thank the reviewer for the constructive comments. In our study, we collected a total of 2,800 Aβ plaques from the postmortem brains of the 2 patients as strict biological replicates (i.e., AD22 and AD27). Specifically, 937, 966, and 897 diffuse, fibrillar, and core plaques were isolated, respectively (Fig. 1B, Table S1). The area of a single plaque typically ranges from 900–1,800 μm^2. For each specific plaque type per biological replicate, we pooled the isolated plaques to achieve a total microdissected area of 0.2 mm^2 (given the 7-μm section thickness, this corresponds to a volume of

roughly 200,000 µm^2 × 7 µm, which is equivalent to the volume of approximately 700 single tissue cells). This pooled input allowed us to reproducibly identify an average of ~3,500 protein groups per sample. Accordingly, we added this information in *Sample preparation for proteomic analysis* (page 19, line 599–601) as follows:

> *"For each specific plaque type per biological replicate, we pooled the isolated plaques to achieve a total microdissected area of 0.2 mm^2 (given the 7-µm section thickness)."*

In addition, Table S1 and Fig EV4 in the original manuscript provide details regarding the numbers of collected plaques and protein groups.

While we fully agree that emerging proteomics tools have enabled single-cell analysis, such ultra-high sensitivity is predominantly achieved in fresh or cultured cells. For example, state-of-the-art workflows analyzing a single cultured mammalian cell (e.g., a HeLa cell) using a timsTOF SCP mass spectrometer with diaPASEF mode can identify ~3,000 protein groups (Brunner et al., PMID: 35226415). However, achieving a comparable proteomic depth in fluorescently stained, formalin-fixed, paraffin-embedded (FFPE) tissue is inherently much more challenging owing to formaldehyde crosslinking and inevitable sample loss during the de-crosslinking processes.

Given these technical constraints and our empirical optimization, pooling a total tissue area of 0.2 mm^2 (i.e., ~700 tissue cell equivalents) is a good balance. It ensures maximal biological discovery depth (i.e., >3,000 protein groups) while maintaining stable sample handling and reliable quantification (Xu et al., PMID: 39572534). More importantly, pathological heterogeneity and intra-patient variability are further addressed by incorporating technical replicates for each individual and controlling for different factors among patients (e.g., sex and *APOE* genotype).

We fully acknowledge that the latest generation of mass spectrometers, such as the Orbitrap Astral or timsTOF Ultra, can capture deep proteomes from much smaller sample inputs—even single tissue cells (Xu et al., PMID: 40240610). Applying these next-generation instruments to dissect intra-patient heterogeneity at true single-plaque resolution is an exciting direction for our future research.

Referee #2 (Comments on Novelty/Model System for Author):

In the manuscript ""DeepPlaque: A scalable multimodal platform for Aβ pathology and brain cell analysis in Alzheimer's" a pipeline was developed to detect and classify Aβ plaques and cells in immunofluorescence images and combine the analysis with other modalities like spatial proteomics. The idea is relevant, the approach is appropriate, the code was published with annotations, and the conclusions seem realistic. The presented application examples of microglia analysis and proteomics consist of only on two cases but the results appear relatively meaningful.

We appreciate the reviewer's thoughtful comments and feedback, which have helped us greatly improve our manuscript. Below are our responses to the specific suggestions and inquiries raised:

Referee #2 (Remarks for Author):

In the manuscript "DeepPlaque: A scalable multimodal platform for Aβ pathology and brain cell analysis in Alzheimer's", the authors developed a pipeline to integrate Amyloid beta and cell image analysis with spatial protein dissection. In detail, the model PlaqueNet was created to classify Amyloid beta plaques in immunofluorescence stains of postmortem human brains from Alzheimer's disease patients into three classes: diffuse, fibrillar, and core plaques. Additionally, microglia cells were automatically detected in six cases. Proteins within the plaques were further analyzed by automated laser microdissection and liquid chromatography-tandem mass spectrometry in two cases. The plaque classification model achieved high performance scores. Microglia were more associated with cored and fibrillar plaques than with diffuse plaques. Integrated protein analysis suggested a high ApoE accumulation and an axonal damage within cored plaques. The methods and results are mostly described in detail and in a comprehensible manner. The code was made publicly available. The following comments might help to improve and the manuscript:

1. How does PlaqueNet react with cerebral amyloid angiopathy (CAA) as input?

We thank the reviewer for the comments. We would like to clarify that CAA was not included in the training dataset for PlaqueNet. Consequently, PlaqueNet is not equipped to detect or classify CAA-related features in the input data.

2. Did you try to train a plaque classification model without the DAPI channel? If yes, did it perform differently?

We sincerely thank the reviewer for this insightful suggestion. We conducted an ablation experiment to train our PlaqueNet model without the DAPI channel (using solely the Aβ plaque target channel) to explicitly evaluate the impact of DAPI on model performance.

With DAPI channel removed, the model's overall detection and segmentation performance decreased significantly to a level almost comparable to the baseline DETR model (please refer to the table below). In addition, the model exhibited noticeable declines in robustness and transferability when evaluated on independent external datasets. These findings confirm that the structural context provided by the DAPI channel is crucial for refining feature discrimination and enhancing the PlaqueNet's generalizability across diverse datasets.

Dataset	Model	mAP	AP50	mDice	AUC
**External test**	**PlaqueNet (with DAPI)**	**0.517**	**0.674**	**0.906**	**0.941**
	PlaqueNet (without DAPI)	0.507	0.661	0.883	0.930
	DETR	0.509	0.658	0.879	0.922
**Transferability test**	**PlaqueNet (with DAPI)**	**0.209**	**0.415**	**0.852**	**0.881**
	PlaqueNet (without DAPI)	0.187	0.381	0.845	0.837
	DETR	0.176	0.364	0.842	0.799

3. The fact "We excluded morphologically ambiguous plaques from classification" should be somehow mentioned in the limitations as this might cause challenges for users when the model is applied on real (messy) data.

We thank the reviewer for the comments. Accordingly, we have included this limitation in the *Discussion* section (page 12, line 350–353) as follows:

> *"Moreover, we excluded morphologically ambiguous plaques from the classification of Aβ plaque species, which may potentially pose challenges in mean average precision, specifically if the images processed by PlaqueNet are not optimal in quality."*

4. Can you add additional information on how the alternative models (YOLOv3, RetinaNet,...) were optimized to get a better impression of how fair the model comparison was?

We thank the reviewer for this constructive feedback. We apologize for any lack of clarity regarding the implementation details of the baseline models in the original manuscript. To ensure a fair comparison with PlaqueNet, we objectively optimized all alternative models. We have supplemented this new information in *Model architecture and implementation details* (page 17, line 511–517) as follows:

> *"When comparing PlaqueNet with other leading object detection models, we implemented all models, including YOLOv3, RetinaNet, Faster R-CNN, DETR, and PlaqueNet, using the widely recognized mmdetection open-source toolbox. We strictly adhered to the highly optimized, architecture-specific, default configurations provided by the mmdetection model zoo. These configurations have been extensively tuned by the community to ensure that each specific architecture reaches its maximum potential."*

5. Despite a small n=6, is there a detectable effect of age, sex or ApoE genotype on the number of microglia per plaque (Fig. 3c)?

We thank the reviewer for the comments. While our study focuses on the development and validation of PlaqueNet for classifying amyloid species in postmortem brains of individual patients, it also serves as a foundational step toward understanding how the microenvironment influences microglial interactions. Indeed, our in-house study alongside published literature suggest that female individuals and those carrying the *APOE4* genotype have more microglia that interact with plaques (Jiang et al., PMID: 37117777). Therefore, it would indeed be compelling to leverage our model to further investigate whether this increase in amyloid-associated microglia is associated with specific plaque species. We hope the reviewer agrees that this exploration represents a valuable pathway for future research. Accordingly, we have added the following new description in the *Discussion* (page 11, line 316–320):

> *"For instance, our group reports that factors, such as sex, APOE genotype, and genetic variants, are associated with the number of microglia interacting with Aβ plaques (58). Therefore, it would be compelling to leverage our model to further investigate if the increase in amyloid-associated microglia is associated with specific Aβ plaque species."*

6. If there is space left, the most relevant results of the microglia and protein analysis might be added in the abstract to convey to readers that these content-related sections are also presented in the manuscript besides the methodology.

We thank the reviewer for the comments. We have revised the *Abstract* (page 2, line 46–50) to include the microglial and protein analysis. This newly added sentence in highlights the findings in the *Results* section about spatial phenotyping (page 7, line 199–202) and proteomic profiling (page 8, line 236–239):

> *"By integrating spatial cellular phenotyping with laser microdissection, DeepPlaque enables high-throughput proteomic analysis of Aβ plaque niches, revealing that microglia are more abundant around core and fibrillar Aβ plaques, with increased expression of apolipoprotein E and amyloid precursor protein in core Aβ plaques."*

7. Fig. 2a: Why is the mean average precision (mAP) of the model 51.7 %? Can you explain a bit more how the score is calculated and what it practically means to have a mAP of 51.7 % when a user applies the model?

We thank the reviewer for the comments. We understand the concern regarding the numerical value of 51.7%. In the field of computer vision, mean average precision (mAP) serves as a comprehensive metric that integrates both detection accuracy and localization precision rather than merely providing a simple percentage of "correct versus incorrect" samples.

To elaborate, mAP is calculated from the precision–recall curve, which reflects how accurately a model identifies objects while ensuring substantial overlap with expert manual annotations (typically requiring an intersection over union [IoU] > 0.5). A score of 51.7% indicates that the model maintains a high confidence level while successfully detecting most targets, even in the complex, low-contrast backgrounds characteristic of biological imaging.

For users applying PlaqueNet, a mAP of 51.7% signifies robust performance (Lin et al., https://doi.org/10.1007/978-3-319-10602-1_48; Stringer et al., PMID: 33318659). This mAP value indicates that the model achieves a high level of accuracy for detecting the 3 types of Aβ plaques and surpasses human interobserver consistency, making it particularly useful in high-throughput screening scenarios in which manual annotation may be unfeasible. Accordingly, we added the following description in the *Results* (page 6, line 162–164):

> *"These results indicate that the model maintains a high confidence level while detecting most targets, even in the complex, low-contrast backgrounds characteristic of biological imaging."*

8. Fig. 3a (left top image): How were the representative regions from whole slide images selected?

We thank the reviewer for the comments. We randomly selected representative regions of interest (ROIs) from the grey matter of the whole-slide images. To minimize bias in the sampling of Aβ plaques, we enabled only the DAPI channel during the random sampling to accurately delineate the grey matter. Accordingly, we have added a new description about this sampling method in *Immunofluorescence staining* (page 14, line 421–423) as follows:

> *"We randomly selected representative regions of interest (ROIs) from the grey matter of the whole-slide images. To minimize bias in the sampling of Aβ plaques, we enabled only the DAPI channel during random sampling to accurately delineate the grey matter."*

9. If not avoided because of a specific reason, the word "disease" might be added in the title: "DeepPlaque: A scalable multimodal platform for Aβ pathology and brain cell analysis in Alzheimer's disease"

We thank the reviewer for the comments. We have now added the word "disease" to the title.

10. Extended Fig. 3: A note in the caption to which class the Abeta plaque belongs to would be convenient.

We thank the reviewer for the comments. Accordingly, we have added a new note in the figure legend of Fig EV3 indicating to which class the Aβ plaque belongs.

11. According to the Data Availability statement "The mass spectrometry dataset is deposited to the ProteomeXchange Consortium via the PRIDE (69) partner repository with the dataset identifier PXD071421.". However, I was not able to find the dataset. Can you double check the identifier and add further information so that it is easier to find it?

We thank the reviewer for the comments. The PRIDE team provides the following details regarding peer review access:

Log in to the PRIDE website using the following details:

> Project accession: PXD071421
>
> Token: 5PnpOMqTA1WT

Alternatively, the reviewer can access the dataset by logging into the PRIDE website using the following account details:

> Username: reviewer_pxd071421@ebi.ac.uk

Password: 1KTtNEKXoSpa

Referee #3 (Comments on Novelty/Model System for Author):

The authors describe an amyloid plaque classification tool that can be used to automate classification and selection of plaques for laser microdissection. This has the potential to be a useful tool to accelerate local amyloid assessments and heterogeneity assessments.

Referee #3 (Remarks for Author):

This study describes a new tool to automate the classification of amyloid plaques and to integrate this classification with selection of plaques for laser capture microdissection to perform MS of local plaque niches. The authors use three classifications: diffuse, fibrillar, and core. The PlaqueNet algorithm exhibited excellent ability to accurately quantify plaques compared to expert humans. When coupled with glial staining and spatial proteomics, the DeepPlaque system was able to identify differences in plaque niches consistent with previous observations (ie differential APOE or homeostatic microglia markers). Overall, this is a promising tool. The study could be strenghtened by a more diverse classification of amyloid heterogeneity, more robust histological staining, and a more thorough description of methods.

We thank the reviewer for the comments. Please see our responses to the major critiques raised:

Major critiques:

1.) The antibodies used to assess different pathologies are not described in the methods.

We thank the reviewer for the comments. As suggested by the reviewer, we have added new information about these details to the *Methods* under *Immunofluorescence staining* (page 13-14, line 410–412):

> *"We continued staining cycles until the final incubation with anti-Aβ antibody (clone NAB228, Santa Cruz Biotechnology, TX, USA), which is commonly used for detecting Aβ in human samples."*

In addition, we have provided the specific clone and concentration of this antibody in Table S4.

2.) The classification of Abeta staining into 3 groups may limit the utility of the tool. For one, the description of a plaque as "diffuse" without the use of a thioflavin-based dye

may reduce accuracy. Second, other forms of amyloid pathology such as coarse-grained (PMID: 32926214), or cotton wool (PMID: 11706935) could be considered.

We thank the reviewer for the comments. Our DeepPlaque system employs immunohistochemical staining with an anti-Aβ antibody, which is widely accepted for detecting Aβ pathology in both research and pathological contexts. Specifically, immunohistochemical staining with an anti-Aβ antibody enables robust multiplex immunostaining of various cell types alongside Aβ pathology. This approach facilitates comprehensive characterization beyond mere plaque identification, allowing for effective quantification of Aβ pathology and its associated microenvironment. In contrast, thioflavin dye-based fluorescence staining is incompatible with most multiplex immunofluorescence staining and imaging protocols.

Accordingly, we added the following sentences to highlight the conceptual novelty in the *Discussion* section (page 11, line 324–329):

> *"In addition, the DeepPlaque system employs immunohistochemical staining with an anti-Aβ antibody, enabling robust multiplex immunostaining of various cell types alongside Aβ pathology. This approach allows for comprehensive cellular characterization beyond mere plaque identification, enabling effective quantification of Aβ pathology and its associated microenvironment."*

To ensure the utility of DeepPlaque, we optimized PlaqueNet to accurately detect and classify only 3 types of Aβ plaques. In addition, we conducted a transferability test to evaluate PlaqueNet's performance using thioflavin-based-stained brain sections; this followed a different immunohistochemical staining protocol, and images were captured with a microscope system different from that used for PlaqueNet development. (Fig. 2E, F, Fig EV2C). In the transferability test, PlaqueNet achieved 73.33% accuracy and an AUC of 0.769 in differentiating diffuse Aβ plaques despite the presence of significant background fluorescence (Fig. 2E, F, Fig EV2C). This confirms PlaqueNet's reliability for applications across different imaging systems and staining protocols; it also enhances its applicability across laboratories. Below are the sentences in the original manuscript that highlight the utility and generalizability of PlaqueNet:

> *"To assess the robustness of PlaqueNet on a new imaging system, we evaluated its performance on a transferability dataset (N = 163 Aβ plaques) acquired with the THUNDER Imager Tissue system, which was not used during PlaqueNet development (**Fig 1B**). While all other models exhibited performance degradation, PlaqueNet maintained an mDice of 0.852 and an overall AUC of 0.881, significantly outperforming the next-best models (i.e., DETR and Faster R-CNN, both p < 0.001 comparing the best-performing model with the second-best model) (**Fig 2B; Fig EV 2B, C**). This confirms PlaqueNet's reliability for applications across different imaging systems."* (*Results*, page 6, line 164–172)

> *“We show that PlaqueNet outperforms state-of-the-art algorithms and expert researchers while maintaining robustness across different imaging systems.”* (*Discussion*, page 10, line 283–285)

Regarding the absence of other plaque types, our study serves as a proof-of-concept to illustrate the integration of a deep learning model with an automated workflow for Aβ plaque species and spatial proteomic profiling of postmortem brain samples in Alzheimer’s disease. Our study specifically focuses on the classification of the more commonly studied diffuse, fibrillar, and core plaques, which are integral to pathological staging in Alzheimer’s disease research in accordance with established criteria from the literature (Dickson et al., PMID: 11483304; Tang et al., PMID: 31092819). We agree that establishing the foundation of the current DeepPlaque system opens the pathway for advancements in detecting and classifying additional Aβ plaque species, including neuritic plaques. However, this endeavor would necessitate retraining the model and collecting postmortem brain samples from familial and early-onset Alzheimer’s disease cases (which have more coarse-grained and cotton wool plaques). We hope the reviewer agrees that this could be a focus for future studies. Nevertheless, we added the following to the *Discussion* (page 12, line 359–362):

> *“While the DeepPlaque system mainly focuses on Aβ plaque species and surrounding cells, we envision its potential to be adapted to examine other types of Aβ plaque species, such as neuritic plaques, as well as other types of proteinopathies in postmortem brain sections.”*

3.) The histological applicability of DeepPlaque is assessed using only P2ry12 staining. Additional approaches could be used to assess the accuracy and utility of DeepPlaque, such as staining for neuritic plaque-tau, which should be absent in diffuse plaques, or a typical DAM marker to show relative increase around fibrillar plaque.

We thank the reviewer for the comments. Regarding the neuritic plaques, which consist of amyloid and p-Tau, our study primarily focused on Aβ-stained plaques. This indicates that we would need to repeat the entire study by incorporating p-Tau staining alongside amyloid staining to retrain the model. The aim of the current study is to provide a proof-of-concept illustrating how AI-based detection and classification of Aβ plaques can be effectively coupled with spatial cellular phenotyping and proteomic profiling of postmortem brain samples from patients with Alzheimer’s disease. We hope the reviewer agrees that this foundational work may serve as a valuable basis for future studies.

Other than staining for homeostatic marker P2ry12, we indeed conducted staining for CD74, a disease-associated microglial marker (Olah et al., PMID: 33257666), in sample AD3 and AD22 as part of our multiplex staining. However, as the absolute numbers of disease-associated microglia expressing CD74 were too small to yield

statistically meaningful comparisons across the 3 Aβ plaque types, we omitted these data from our manuscript.

10th Jun 2026

Dear Prof. Ip,

Thank you for the submission of your revised manuscript to EMBO Molecular Medicine. We have now received the enclosed reports from the two referees who were asked to re-evaluate your manuscript. As you will see, while Referee #2 is supportive of publication, Referee #3 raised concerns regarding the broader applicability of the proposed method.

In light of these comments, and given that Referee #3 still considers the method to be promising, we would like to invite you to submit a minor revision. Specifically, we ask that you more explicitly acknowledge these points raised by Referee #3 as limitations of the current study and discuss them as potential directions for future work.

On a more editorial level:

1. Remove "Author contribution" section from the manuscript file.

2. Data availability: Please remove the reviewer token and ensure that all datasets are publicly available upon acceptance of the manuscript. In addition, please provide a specific URL for the deposited dataset. The Code Availability statement should be incorporated into the Data Availability section, and the separate Code Availability heading should be removed.

3. Please resolve the following author name discrepancy: Amy K. Y. Fu in the manuscript versus Amy Fu in the submission system.

4. Please provide the email addresses of all corresponding authors on the title page of the manuscript.

5. The supplementary Excel file containing Tables S1-S5 is relatively complex and should be converted into EV Datasets. Please upload the tables separately as Dataset EV1 to EV5. The nomenclature should be updated consistently throughout the submission, including: source file names, titles in the submission system, callouts in the manuscript.

6. The references need to be formatted according to the EMBO Molecular Medicine reference style. Please list up to 10 co-authors of a paper before adding et al. in the reference list. Citations should be listed in alphabetical order. DOIs should only be used for preprints and datasets that have not been published yet.

7. Please add a Disclosure Statement and Competing Interests section. Please note that employment by a biotechnology company must be explicitly disclosed in the Declaration of Interests and Competing Interests Statement.

8. Please download and fill our Reagents and Tools Table template (.docx), which you can find in our author guidelines: https://link.springer.com/journal/44320/submission-guidelines#structuredmethods.

When submitting your revised manuscript, please do not include the Reagents and Tools Table in the Methods section of the manuscript but upload it as a separate file choosing the file type "Reagent Table".

9. Please provide "The paper explained" in the manuscript file: EMBO Molecular Medicine articles are accompanied by a summary of the articles to emphasize the major findings in the paper and their medical implications for the non-specialist reader. Please provide a draft summary of your article highlighting

- the medical issue you are addressing,

- the results obtained and

- their clinical impact.

This may be edited to ensure that readers understand the significance and context of the research. Please refer to any of our published articles for an example.

10. Please provide a Synopsis as a separate Word document to further enhance discoverability. Synopses are displayed on the journal webpage and are freely accessible to all readers. They include a short stand first (maximum of 300 characters, including space) as well as 2-5 one-sentences bullet points that summarizes the paper. Please write the bullet points to summarize the key NEW findings. They should be designed to be complementary to the abstract - i.e. not repeat the same text. We encourage inclusion of key acronyms and quantitative information (maximum of 30 words / bullet point). Please use the passive voice. Please attach these in a separate file or send them by email, we will incorporate them accordingly.

11. Please also provide a visual abstract as a separate PNG file with dimensions of exactly 550 px (width) × 300 px (height) to highlight the paper on our homepage.

12. "Supplementary Method" is not allowed. Please move this content to the main Methods section and remove the callout.

13. Please resolve the following discrepancies between the source data provided and the Source Data Checklist:
- 1A is listed on the checklist, but source data for 1C was provided.
- 3A is listed on the checklist, but source data for 3B and 3D were provided.

14. Please address the following issues in figure legends:
- Please note that the exact p values are not provided in the legends of 2A,B; 3C,E,F,G; 4B-D.
- Please note that scale bar and its definition are missing for figure EV-1C

15. "Acknowledgments" should be placed after the "Data availability" section and before "Discussion" section.

I look forward to reading a new revised version of your manuscript as soon as possible.

Kind regards,
Jingyi

Jingyi Hou
Senior Editor
EMBO Molecular Medicine

*** Instructions to submit your revised manuscript ***

*** PLEASE NOTE *** As part of the EMBO Publications transparent editorial process initiative (see our Editorial at https://www.embopress.org/doi/pdf/10.1002/emmm.201000094), EMBO Molecular Medicine will publish online a Review Process File to accompany accepted manuscripts.

In the event of acceptance, this file will be published in conjunction with your paper and will include the anonymous referee reports, your point-by-point response and all pertinent correspondence relating to the manuscript. If you do NOT want this file to be published, please inform the editorial office at contact@embomolmed.org.

*****

When preparing your revised manuscript, please refer to our guidelines: https://link.springer.com/journal/44321/submission-guidelines#cms-Revised-submissions. We perform an initial quality control of all revised manuscripts before re-review; failure to include requested items will delay the evaluation of your revision.

***** Reviewer's comments *****

Referee #2 (Comments on Novelty/Model System for Author):

As given in the previous review

Referee #2 (Remarks for Author):

I am satisfied with the author's responses

Referee #3 (Comments on Novelty/Model System for Author):

The method described here seems promising, but appears to be only applicable to a very defined set of staining and imaging conditions.

Referee #3 (Remarks for Author):

The authors have revised their previous study. Overall, the method remains promising, but it seems fairly inflexible beyond what is described in the paper, which lessens the potential impact and utility.

Re: EMM-2026-23599-T

Title: DeepPlaque: A scalable multimodal platform for Aβ pathology and cell analysis in Alzheimer's disease

Point-by-point responses to reviewers:

Referee #2 (Comments on Novelty/Model System for Author):

As given in the previous review

Referee #2 (Remarks for Author):

I am satisfied with the author's responses

We thank the reviewer for the comment.

Referee #3 (Comments on Novelty/Model System for Author):

The method described here seems promising, but appears to be only applicable to a very defined set of staining and imaging conditions.

Referee #3 (Remarks for Author):

The authors have revised their previous study. Overall, the method remains promising, but it seems fairly inflexible beyond what is described in the paper, which lessens the potential impact and utility.

We thank the reviewer for this valuable comment. Our current work establishes a crucial foundation for the characterization of amyloid plaque species. This model offers a versatile platform that can be further developed to analyze other amyloid plaque types, such as neuritic plaques, and to explore different proteinopathies. This adaptability will significantly contribute to understanding the impact of amyloid pathology on neighboring brain cells. We have elaborated on these potential future avenues and acknowledged the limitations of the present study in the Discussion (page 13-14, lines 405-416):

"Building on this foundation, we envision our DeepPlaque system could be further adapted to examine other types of Aβ plaque species, such as neuritic plaques, by incorporating the immunostaining of phosphorylated tau proteins. This adaptability can also extend to exploring other proteinopathies in postmortem brain sections, thereby broadening the system's utility in neurodegenerative disease research and enhancing our understanding of how amyloid pathology impacts neighboring brain cells."

"Moreover, as the first proof-of-concept study, our PlaqueNet addresses the classification of the more commonly studied Aβ plaque types—diffuse, fibrillar, and core plaques—which are essential for pathological staging in Alzheimer’s disease research. While the current version focuses on these Aβ plaque types, the underlying architecture has been developed with extensibility in mind, enabling the ready incorporation of additional species, such as neuritic plaques, in future updates.”

2nd Revision - Editorial Decision 6th Jul 2026

6th Jul 2026

Dear Prof. Ip,

We are pleased to inform you that your manuscript is accepted for publication and is now being sent to our publisher to be included in the next available issue of EMBO Molecular Medicine.

Your manuscript will be processed for publication by EMBO Press. It will be copy edited and you will receive page proofs prior to publication. Please note that you will be contacted by Springer Nature Author Services to complete licensing and payment information.

You may qualify for financial assistance for your publication charges - either via a Springer Nature fully open access agreement or an EMBO initiative. Check your eligibility: https://link.springer.com/journal/44321/how-to-publish-with-us

Should you be planning a Press Release on your article, please notify the production team via this form in order to coordinate publication and release dates: https://forms.cloud.microsoft/e/eH4A8JVP6g

If you have any questions, please do not hesitate to contact the Editorial Office. Thank you for your contribution to EMBO Molecular Medicine.

Sincerely,
Jingyi

Jingyi Hou
Senior Editor
EMBO Molecular Medicine

>>> Please note that it is EMBO Molecular Medicine policy for the transcript of the editorial process (containing referee reports and your response letter) to be published as an online supplement to each paper. If you do NOT want this, you will need to inform the Editorial Office via email immediately. More information is available here: https://link.springer.com/partners/embo-press/editorial-policies#Peer%20review

EMBO Press Author Checklist

Corresponding Author Name:Nancy Ip	
Journal Submitted to: EMBO Molecular Medicine	
Manuscript Number: EMM-2026-23599-T	

USEFUL LINKS FOR COMPLETING THIS FORM
The EMBO Journal - Author Guidelines
EMBO Reports - Author Guidelines
Molecular Systems Biology - Author Guidelines
EMBO Molecular Medicine - Author Guidelines

Reporting Checklist for Life Science Articles (updated January
This checklist is adapted from Materials Design Analysis Reporting (MDAR) Checklist for Authors. MDAR establishes a minimum set of requirements in transparent reporting in the life sciences (see Statement of Task: 10.31222/osf.io/9sm4x). Please follow the journal's guidelines in preparing your
Please note that a copy of this checklist will be published alongside your article.

Abridged guidelines for figures
1. Data
The data shown in figures should satisfy the following conditions:
- the data were obtained and processed according to the field's best practice and are presented to reflect the results of the experiments in an accurate and unbiased manner.
- ideally, figure panels should include only measurements that are directly comparable to each other and obtained with the same assay.
- plots include clearly labeled error bars for independent experiments and sample sizes. Unless justified, error bars should not be shown for technical
- if n<5, the individual data points from each experiment should be plotted. Any statistical test employed should be justified.
- Source Data should be included to report the data underlying figures according to the guidelines set out in the authorship guidelines on Data

2. Captions
Each figure caption should contain the following information, for each panel where they are relevant:
- a specification of the experimental system investigated (eg cell line, species name).
- the assay(s) and method(s) used to carry out the reported observations and measurements.
- an explicit mention of the biological and chemical entity(ies) that are being measured.
- an explicit mention of the biological and chemical entity(ies) that are altered/varied/perturbed in a controlled manner.
- the exact sample size (n) for each experimental group/condition, given as a number, not a range;
- a description of the sample collection allowing the reader to understand whether the samples represent technical or biological replicates (including how many animals, litters, cultures, etc.).
- a statement of how many times the experiment shown was independently replicated in the laboratory.
- definitions of statistical methods and measures:
  - common tests, such as t-test (please specify whether paired vs. unpaired), simple χ2 tests, Wilcoxon and Mann-Whitney tests, can be unambiguously identified by name only, but more complex techniques should be described in the methods section;
  - are tests one-sided or two-sided?
  - are there adjustments for multiple comparisons?
  - exact statistical test results, e.g., P values = x but not P values < x;
  - definition of 'center values' as median or average;
  - definition of error bars as s.d. or s.e.m.

Please complete ALL of the questions below.
Select "Not Applicable" only when the requested information is not relevant for your study.

Materials

Newly Created Materials	Information included in the manuscript?	In which section is the information available? (Reagents and Tools Table, Materials and Methods, Figures, Data Availability Section)
New materials and reagents need to be available; do any restrictions apply?	Yes	Materials and Methods, Data Availability

Antibodies	Information included in the manuscript?	In which section is the information available? (Reagents and Tools Table, Materials and Methods, Figures, Data Availability Section)
For **antibodies** provide the following information:<br>- Commercial antibodies: RRID (if possible) or supplier name, catalogue number and or/clone number<br>- Non-commercial: RRID or citation	Yes	Materials and Methods, Reagents and Tools table, Dataset EV4

DNA and RNA sequences	Information included in the manuscript?	In which section is the information available? (Reagents and Tools Table, Materials and Methods, Figures, Data Availability Section)
**Short novel DNA or RNA including primers, probes**: provide the sequences.	Not Applicable	

Cell materials	Information included in the manuscript?	In which section is the information available? (Reagents and Tools Table, Materials and Methods, Figures, Data Availability Section)
**Cell lines:** Provide species information, strain. Provide accession number in repository **OR** supplier name, catalog number, clone number, and/**OR** RRID.	Not Applicable	
**Primary cultures:** Provide species, strain, sex of origin, genetic modification status.	Not Applicable	
Report if the cell lines were recently **authenticated** (e.g., by STR profiling) and tested for mycoplasma contamination.	Not Applicable	

Experimental animals	Information included in the manuscript?	In which section is the information available? (Reagents and Tools Table, Materials and Methods, Figures, Data Availability Section)
**Laboratory animals or Model organisms:** Provide species, strain, sex, age, genetic modification status. Provide accession number in repository **OR** supplier name, catalog number, clone number, **OR** RRID.	Not Applicable	
**Animal observed in or captured from the field:** Provide species, sex, and age where possible.	Not Applicable	
Please detail **housing and husbandry conditions**.	Not Applicable	

Plants and microbes	Information included in the manuscript?	In which section is the information available? (Reagents and Tools Table, Materials and Methods, Figures, Data Availability Section)
**Plants:** provide species and strain, ecotype and cultivar where relevant, unique accession number if available, and source (including location for collected wild specimens).	Not Applicable	
**Microbes:** provide species and strain, unique accession number if available, and source.	Not Applicable	

Human research participants	Information included in the manuscript?	In which section is the information available? (Reagents and Tools Table, Materials and Methods, Figures, Data Availability Section)
If collected and within the bounds of privacy constraints report on age, sex and gender or ethnicity for all study participants.	Yes	Dataset EV3

Core facilities	Information included in the manuscript?	In which section is the information available? (Reagents and Tools Table, Materials and Methods, Figures, Data Availability Section)
If your work benefited from core facilities, was their service mentioned in the acknowledgments section?	Yes	Acknowledgments

Design

Study protocol	Information included in the manuscript?	In which section is the information available? (Reagents and Tools Table, Materials and Methods, Figures, Data Availability Section)
If study protocol has been **pre-registered, provide DOI in the manuscript**. For clinical trials, provide the trial registration number **OR** cite DOI.	Not Applicable	
Report the **clinical trial registration number** (at ClinicalTrials.gov or equivalent), where applicable.	Not Applicable	

Laboratory protocol	Information included in the manuscript?	In which section is the information available? (Reagents and Tools Table, Materials and Methods, Figures, Data Availability Section)
Provide DOI OR other citation details if **external detailed step-by-step protocols** are available.	Not Applicable	

Experimental study design and statistics	Information included in the manuscript?	In which section is the information available? (Reagents and Tools Table, Materials and Methods, Figures, Data Availability Section)
Include a statement about **sample size** estimate even if no statistical methods were used.	Not Applicable	
Were any steps taken to minimize the effects of subjective bias when allocating animals/samples to treatment (e.g. **randomization procedure**)? If yes, have they been described?	Not Applicable	
Include a statement about **blinding** even if no blinding was done.	Not Applicable	
Describe **inclusion/exclusion criteria** if samples or animals were excluded from the analysis. Were the criteria pre-established?<br><br>If sample or data points were omitted from analysis, report if this was due to attrition or intentional exclusion and provide justification.	Not Applicable	
For every figure, are **statistical tests** justified as appropriate? Do the data meet the assumptions of the tests (e.g., normal distribution)? Describe any methods used to assess it. Is there an estimate of variation within each group of data? Is the variance similar between the groups that are being statistically compared?	Yes	Materials and Methods

Sample definition and in-laboratory replication	Information included in the manuscript?	In which section is the information available? (Reagents and Tools Table, Materials and Methods, Figures, Data Availability Section)
In the figure legends: state number of times the experiment was **replicated** in laboratory.	Not Applicable	
In the figure legends: define whether data describe **technical or biological replicates**.	Yes	Materials and Methods, Figure EV legends

Ethics

Ethics	Information included in the manuscript?	In which section is the information available? (Reagents and Tools Table, Materials and Methods, Figures, Data Availability Section)
Studies involving **human participants**: State details of **authority granting ethics approval** (IRB or equivalent committee(s), provide reference number for approval.	Yes	Materials and Methods
Studies involving **human participants**: Include a statement confirming that **informed consent** was obtained from all subjects and that the experiments conformed to the principles set out in the WMA Declaration of Helsinki and the Department of Health and Human Services Belmont Report.	Yes	Materials and Methods
Studies involving **human participants: For publication of patient photos**, include a statement confirming that consent to publish was obtained.	Not Applicable	
Studies involving experimental **animals**: State details of **authority granting ethics approval** (IRB or equivalent committee(s), provide reference number for approval. Include a statement of compliance with ethical regulations.	Not Applicable	
Studies involving **specimen and field samples:** State if relevant **permits** obtained, provide details of authority approving study; if none were required, explain why.	Not Applicable	

Dual Use Research of Concern (DURC)	Information included in the manuscript?	In which section is the information available? (Reagents and Tools Table, Materials and Methods, Figures, Data Availability Section)
Could your study fall under dual use research restrictions? Please check biosecurity documents and list of **select agents and toxins** (CDC): https://www.selectagents.gov/sat/list.htm	Not Applicable	
If you used a select agent, is the security level of the lab appropriate and reported in the manuscript?	Not Applicable	
If a study is subject to dual use research of concern regulations, is the name of the **authority granting approval and reference number** for the regulatory approval provided in the manuscript?	Not Applicable	

Reporting

The MDAR framework recommends adoption of discipline-specific guidelines, established and endorsed through community initiatives. Journals have their own policy about requiring specific guidelines and recommendations to complement MDAR.

Adherence to community standards	Information included in the manuscript?	In which section is the information available? (Reagents and Tools Table, Materials and Methods, Figures, Data Availability Section)
State if relevant guidelines or checklists (e.g., **ICMJE, MIBBI, ARRIVE, PRISMA**) have been followed or provided.	Not Applicable	
For **tumor marker prognostic studies,** we recommend that you follow the **REMARK** reporting guidelines (see link list at top right). See author guidelines, under 'Reporting Guidelines'. Please confirm you have followed these guidelines.	Not Applicable	
For **phase II and III randomized controlled trials**, please refer to the **CONSORT** flow diagram (see link list at top right) and submit the CONSORT checklist (see link list at top right) with your submission. See author guidelines, under 'Reporting Guidelines'. Please confirm you have submitted this list.	Not Applicable	

Data Availability

Data availability	Information included in the manuscript?	In which section is the information available? (Reagents and Tools Table, Materials and Methods, Figures, Data Availability Section)
Have **primary datasets** been deposited according to the journal's guidelines (see 'Data Deposition' section) and the respective accession numbers provided in the Data Availability Section?	Yes	Data Availability
Were **human clinical and genomic datasets** deposited in a public access-controlled repository in accordance to ethical obligations to the patients and to the applicable consent agreement?	Not Applicable	
Are **computational models** that are central and integral to a study available without restrictions in a machine-readable form? Were the relevant accession numbers or links provided?	Yes	Data Availability
If publicly available data were reused, provide the respective **data citations in the reference list**.	Not Applicable	
